# EPIKOL, a chromatin-focused CRISPR/Cas9-based screening platform, to identify cancer-specific epigenetic vulnerabilities

**DOI:** 10.1038/s41419-022-05146-4

**Published:** 2022-08-16

**Authors:** Ozlem Yedier-Bayram, Bengul Gokbayrak, Alisan Kayabolen, Ali Cenk Aksu, Ayse Derya Cavga, Ahmet Cingöz, Ezgi Yagmur Kala, Goktug Karabiyik, Rauf Günsay, Beril Esin, Tunc Morova, Fırat Uyulur, Hamzah Syed, Martin Philpott, Adam P. Cribbs, Sonia H. Y. Kung, Nathan A. Lack, Tamer T. Onder, Tugba Bagci-Onder

**Affiliations:** 1 Koç University Research Center for Translational Medicine (KUTTAM), Istanbul, Türkiye; 2 Biostatistics, Bioinformatics and Data Management Core, KUTTAM, Istanbul, Türkiye; 3 Koç University School of Medicine, Istanbul, Türkiye; 4 Vancouver Prostate Centre, University of British Columbia, Vancouver, BC Canada; 5 Koç University Department of Computational Biology, Istanbul, Türkiye; 6 Botnar Research Centre, Nuffield Department of Orthopedics, Rheumatology and Musculoskeletal Sciences, University of Oxford, Oxford, UK

**Keywords:** Breast cancer, Epigenetics, Transcriptomics

## Abstract

Dysregulation of the epigenome due to alterations in chromatin modifier proteins commonly contribute to malignant transformation. To interrogate the roles of epigenetic modifiers in cancer cells, we generated an epigenome-wide CRISPR-Cas9 knockout library (EPIKOL) that targets a wide-range of epigenetic modifiers and their cofactors. We conducted eight screens in two different cancer types and showed that EPIKOL performs with high efficiency in terms of sgRNA distribution and depletion of essential genes. We discovered novel epigenetic modifiers that regulate triple-negative breast cancer (TNBC) and prostate cancer cell fitness. We confirmed the growth-regulatory functions of individual candidates, including SS18L2 and members of the NSL complex (KANSL2, KANSL3, KAT8) in TNBC cells. Overall, we show that EPIKOL, a focused sgRNA library targeting ~800 genes, can reveal epigenetic modifiers that are essential for cancer cell fitness under in vitro and in vivo conditions and enable the identification of novel anti-cancer targets. Due to its comprehensive epigenome-wide targets and relatively high number of sgRNAs per gene, EPIKOL will facilitate studies examining functional roles of epigenetic modifiers in a wide range of contexts, such as screens in primary cells, patient-derived xenografts as well as in vivo models.

## Introduction

Epigenetic modifications regulate gene expression and are altered by developmental and environmental cues [1]. Strict epigenetic control is required during embryogenesis, differentiation, cell fate decisions, and maintenance of cell identity [2]. Dysregulation of the epigenome has emerged as an important mechanism contributing to various pathologies including tumorigenesis. Epigenome-level alterations pave the way for pre-malignant cells to acquire cancer hallmarks including aggressiveness, environmental adaptation, and resistance to therapy [3]. For example, in many cancer types, DNA hypomethylation or aberrant histone acetylation activates proto-oncogene expression, whereas DNA or histone hypermethylation represses tumor-suppressor expression [4–8]. Recent cancer genome sequencing studies revealed mutations in many epigenetic modifiers that are associated with various cancers [9], such as DNMT3A in acute myeloid leukemia [10, 11], IDH1/2 in glioblastoma [12, 13], CREBBP/EP300 in small-cell lung cancer [14] and ARID1A in gastric cancer [15]. These driver mutations are thought to act in part by increasing cellular plasticity during development of malignant tumors. Given this critical role, small molecule inhibitors targeting epigenetic regulators are promising anti-cancer drugs and have shown efficacy in various cancer types [16]. However, first-generation molecules have had limited clinical benefit due to high toxicity [17, 18]. To overcome these limitations, newer molecules are being developed and tested in clinical trials [19–21]. Exploiting synthetic lethal interactions between epigenetic modifiers via small molecule inhibitors is a promising therapeutic approach to target the disease in a cancer-specific manner [22–24].

CRISPR/Cas9 technology is a fast, effective, and easy-to-use genome engineering method and has drastically accelerated functional genomics research [25]. Its simplicity allows for the generation of multiplexed sgRNA libraries to interrogate gene functions in pooled genome-wide knockout screens [26, 27]. Negative selection screens identified essential genes in different contexts [28–30], while positive selection screens helped to identify ‘winner’ genes under a given selective pressure [26, 27]. Although genome-wide CRISPR knockout libraries are versatile tools to study various phenotypes simultaneously, the design and execution of such experiments are laborious and expensive. Frequently, secondary screens focusing on the pathways identified in the primary screen are performed to eliminate false-negative results and obtain high confidence leads. Unlike the limited number of sgRNAs per gene in genome-wide libraries, sgRNA numbers per gene can be increased in focused libraries to enhance the reliability of the observed phenotype [31]. Therefore, focused sgRNA libraries have emerged as a way to overcome these challenges by reducing the cost and labor and maximizing the yield and signal/noise ratio [32]. In addition, focused libraries may be advantageous in experimental systems that require clinically relevant models such as primary cells, patient-derived xenografts [33] or in vivo models [34–36]. To date, various focused libraries have been generated targeting microRNAs [37], kinases [38, 39], nuclear proteins [33], epigenetic modifiers [40–42] or genes belonging to a certain pathway such as DNA-damage response [43].

Here, we present a focused Epigenetic Knockout Library (EPIKOL), which targets a broader range of epigenetic modifiers and consists of more sgRNAs for each gene when compared to previously published libraries [40–42]. Utilizing this library in in vitro screens of two different cancer types, we revealed novel epigenetic modifiers that regulate cancer cell fitness. We validated several of these genes in TNBCs, demonstrating the suitability of the library for the identification of epigenetic vulnerabilities of cancer cells. We also performed an in vivo screen with EPIKOL and identified SS18L2, a previously uncharacterized gene, as a cell cycle regulator under both in vitro and in vivo conditions.

## Methods

### Library content of EPIKOL

To generate a customized Epigenetic Knockout Library (EPIKOL), curated epigenetic modifiers in the EpiFactors database were targeted by sgRNAs [44]. In addition to 719 genes that have roles in chromatin-related pathways, 25 genes from different families (nuclear receptors, ABC transporters, apoptosis or metastasis-related proteins) were included to serve as internal controls in specific screen setups. 35 essential genes, such as ribosomal protein-encoding genes and 80 non-targeting sgRNAs were also included. 35 essential genes were determined through analysis of publicly available screen data of 60 different cell lines obtained from the Genome CRISPR database [45]. Among them, genes that have the highest Log2FC were included in the library. Each gene in EPIKOL is targeted by 10 sgRNAs that were chosen from previously established genome-wide CRISPR knockout libraries [46, 47]. Additional sgRNAs were designed by using CCTop and E-CRISP tools in cases where the total number of sgRNAs did not reach 10 per gene due to overlapping sequences in existing libraries [48]. Genes and sequences of sgRNAs of EPIKOL are available in Supplementary Table 1. Details of library cloning, and sequencing can be found in Supplementary Information.

### In vitro CRISPR screens

Cas9-expressing stable cell lines were generated by transducing the cells with LentiCas9-blast virus at MOI = 1 for TNBC and MOI = 5 for PCa cell lines. Cells were selected with blasticidin for 5 days and maintained in blasticidin-containing media for several passages prior to library infection. Negative selection screens with EPIKOL were performed as three biological replicates. Cells were transduced with EPIKOL at low MOI = (0.3–0.4) with 1000× coverage for TNBC and 500x coverage for PCa in the presence of 8 μg/ml protamine sulfate. Following 3 days of puromycin selection, cells were collected (8 × 10^6^cells for TNBC, 4 × 10^6^ for PCa) to serve as a reference point for baseline sgRNA distribution. The remaining cells were kept in culture for 15–16 population doublings. At the end of each screen, cells were collected (8 × 10^6^cells for TNBC, 4 × 10^6^ for PCa) and stored at −80 ^o^C until genomic DNA isolation. Details of next-generation sequencing, and analysis of screen results can be found in Supplementary Information.

### Dual-color competition assays

For validation of EPIKOL screen candidate hits, dual color competition assays were performed. Cas9-stable cells were transduced with either PGK-H2BmCherry (Addgene #21217) or PGK-H2BeGFP (Addgene #21210) viruses at high MOI ~5 to make sure every cell was fluorescently labeled. 50,000 cells were seeded in 12 well-plates, mCherry+ cells were transduced with LentiGuide-NT1 viruses, while eGFP+ cells were transduced with viruses carrying sgRNA-X for selected genes. For each gene, two different sgRNAs were used (Supplementary Table 2). After 16 h, viral media were changed with fresh media and next day puromycin selection was started. After 3 days of puromycin selection, mCherry+ and eGFP+ cells were mixed in a 1:1 ratio and re-seeded into 24-well plates in triplicates. One day after seeding, Day0 measurements were taken by acquiring 3 × 3 images with a 4× objective in Cytation5 (BioTek, USA). Cells were incubated for the subsequent 16 days, and images were taken at Day4, Day8, Day12, and Day16. Number of mCherry+ and eGFP+ cells were counted from images using Gen5 software (BioTek, USA) and each measurement was normalized to Day0 to determine the percentage of eGFP+ cells.

### In vivo CRISPR screen

All in vivo experiments were approved by Koç University Animal Experiments Ethics Committee. Cas9-expressing MDA-MB-231 cells were transduced with Firefly Luciferase (Fluc) expressing lentiviruses. Cells were transduced with EPIKOL at low MOI = (0.3–0.4) with 1000× coverage in the presence of 8 µg/ml protamine sulfate. Following three days of puromycin selection, an initial timepoint pellet was collected as 8 × 10^6^ cells. Remaining cells in DMEM-10% FBS were mixed with Matrigel (354277, Corning) in 1:1 ratio aiming for 8 × 10^6^ cells in total of 150 µl per injection. Six 8-weeks old Nude mice were used. Tumor cells were injected subcutaneously into both flanks of each mouse and monitored using IVIS Lumina III (Perkin Elmer, USA) weekly following intraperitoneal 150 μg/g body weight of D-Luciferin injection. At 2- and 4-weeks post-injection, three mice were sacrificed, and tumors were fresh-frozen in liquid nitrogen. Whole tumors were grinded using pestles. Genomic DNAs from homogenized tumors were isolated using NucleoSpin Tissue kit as described above. Nested PCRs for library amplification of three out of six tumors for each timepoint were performed as described above. 16.5 µg gDNA was used per tumor to account for the gDNA that might be coming from the basement membrane that is covering the tumors. Details of next-generation sequencing and analysis of screen results can be found in Supplementary Information.

### In vivo experiments for validation

MDA-MB-231-Cas9-Fluc cells were infected with either NT1 or SS18L2 sgRNA carrying viruses and selected with puromycin for 3-4 days. After selection, cells were mixed with Matrigel (354277, Corning) in a 1:1 ratio aiming for 4 × 10^6^ cells in a total of 100 µl per injection. Eight Nude mice were injected subcutaneously with tumors carrying NT1 and SS18L2 sgRNA in the left and right flank, respectively. Tumor growth was monitored using IVIS Lumina III (Perkin Elmer, USA) weekly following intraperitoneal 150 μg/g body weight of D-Luciferin injection.

### RNA sequencing

MDA-MB-231-Cas9 cells were infected with either NT1 or SS18L2 sgRNA encoding lentiviruses and selected with puromycin for 3–4 days. Cell pellets were collected as triplicates at day 6 post-transduction. Details of RNA library preparation and sequencing can be found in Supplementary Information.

### PIP-FUCCI cell cycle analysis

pLenti-CMV-Blast-PIP-FUCCI (Addgene #138715) plasmid was used for the analysis of cell cycle transitions. MDA-MB-231-Cas9 cells were infected with PIP-FUCCI viruses with high MOI since blasticidin selection would not be applicable. Then, cells were infected with NT1 or SS18L2 sgRNA carrying viruses and selected with puromycin. At day 3 post-transduction, cells were seeded to 6-well plates. From day 4 to day 8, phase contrast and fluorescent images were taken as 2 × 2 with 10× objectives using Cytation5 (Biotek, USA). Cells expressing PCNA-interacting protein (PIP) degron appeared as green due to fused mVenus fluorescent protein during G1 and G2/M phases [49]. On the other hand, cells expressing mCherry-Gem_1-110_ appeared as red with increasing intensity during S and G2/M phases. During G2 phase, overlapping double-positive signals were more nuclear while during the M phase the cells had both fluorescent signals more diffusely expressed due to the disassembly of the nuclear envelope. G2/M-arrested cells were quantified by counting the mCherry+ cells in mVenus+ population and presented as the ratio of mVenus+ population.

### Statistical analysis

Analysis of EPIKOL data was performed by using the Robust Rank Aggregation (RRA) method in MAGeCK. Unless otherwise stated, *P* values were determined by two-tailed Student’s *t*-test for all experiments in GraphPad Prism8, *P < 0.05, **P < 0.01, ***P < 0.001.

### Note

Information on cell culture, western blotting, quantitative RT-PCR, virus production, clonogenic assays, cell cycle and apoptosis assays, and immunohistochemistry are given in Supplementary Information.

## Results

### Generation of EPIKOL and library performance in multiple cancer cell lines

**Fig. 1:**
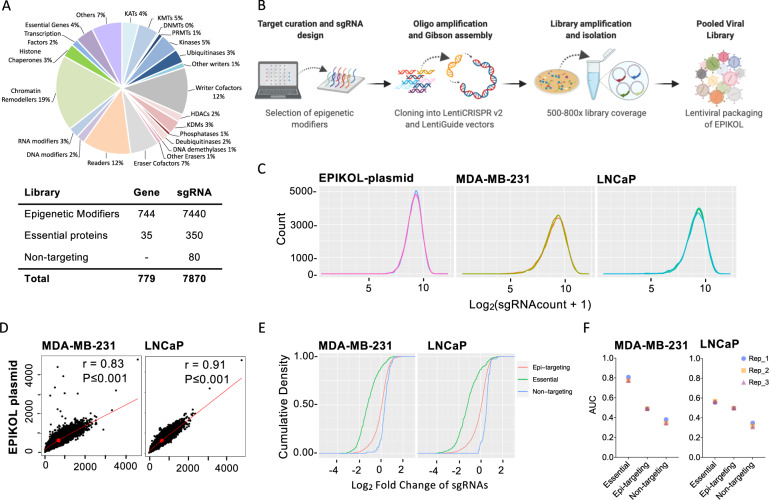
Focused Epigenetic knock-out library (EPIKOL) generation and quality check. **A** Composition of EPIKOL library and number of sgRNAs/gene. **B** Steps of library generation. Figure created with BioRender.com **C** sgRNA density plots from LentiGuide plasmid containing EPIKOL and MDA-MB-231 or LNCaP cells infected with EPIKOL virus. Cell pellets collected after puromycin selection were used for the cell lines. **D** Correlation analysis of plasmid library and samples from EPIKOL-infected cells at initial timepoints. **E** Cumulative density plots showing differential depletion of sgRNAs targeting essential genes when compared to non-targeting sgRNAs. **F** Comparison of Area Under the Curve (AUC) for sgRNAs targeting essential genes, epigenetic modifiers and sgRNAs that are non-targeting. Representative plots of cells screened with EPIKOL for ~15 population doublings were shown.

To study the effect of epigenetic modifiers in multiple cancer types, we generated an epigenome-wide pooled CRISPR library. Epigenetic Knockout Library (EPIKOL) includes 7870 sgRNAs targeting 719 epigenetic modifiers, 25 context-specific controls and 35 pan-essential genes along with 80 non-targeting controls (Fig. 1A, B) in two different lentiviral backbones. Both the plasmid pool and library-transduced cells were sequenced to confirm library complexity and sgRNA distribution (Fig. S1A, Fig. 1C). sgRNA representations between the plasmid pool and transduced cell lines at the initial timepoint of the screens were highly correlated (*R* = 0.83 for MDA-MB-231, *R* = 0.91 for LNCaP), indicating that no bias was introduced during cloning or transduction steps (Fig. 1D, Fig. S1B). To evaluate the efficacy of the screens, we compared the depletion scores of epigenetic-targeted genes versus essential genes and non-targeting controls. We observed significant depletion upon knockout of essential genes and no change in non-targeting controls (Fig. 1E, Fig. S1C).

Library performance was evaluated by calculating the area under the curve (AUC) for sgRNAs targeting essential genes and non-targeting controls. In multiple cell lines, essential gene targeting sgRNAs had AUC > 0.5 indicating that these genes were preferentially depleted, whereas non-targeting gRNAs had AUC < 0.5 indicating their stationary behavior (Fig. 1F, Fig. S1D) [34]. Altogether, these initial analyses demonstrated that EPIKOL preserves normal distribution of sgRNAs both in plasmids and infected cells, and functions as expected in depletion screens.

### EPIKOL screens revealed epigenetic vulnerabilities of TNBC and prostate cancer cell lines

**Fig. 2:**
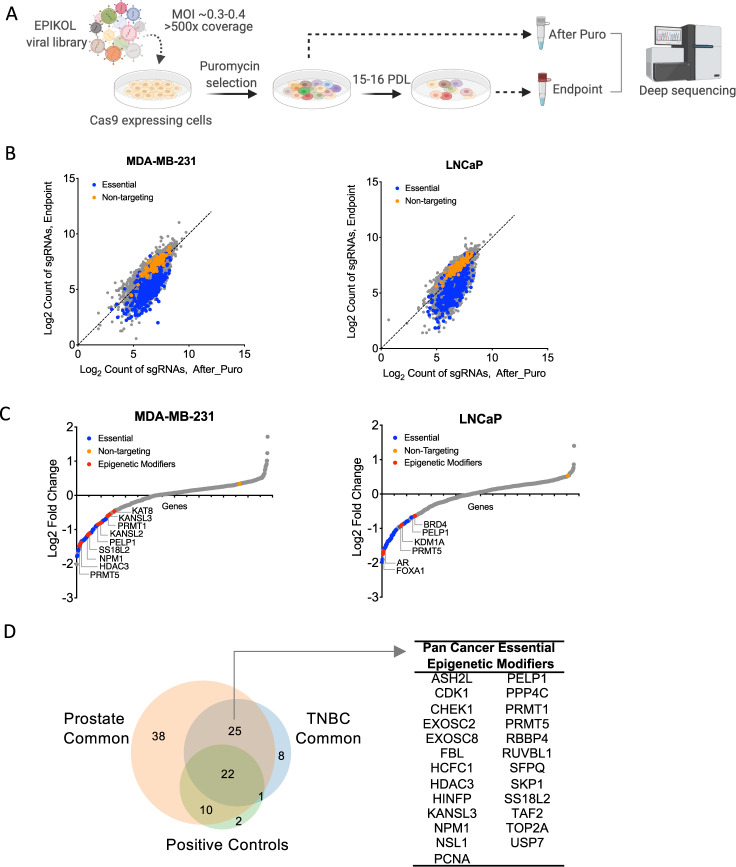
EPIKOL screens on TNBC and prostate cancer cell lines revealed cancer-specific and pan-cancer epigenetic modifiers that regulate cell fitness. **A** Summary of screening procedure. Figure created with BioRender.com **B** Log2 counts of sgRNAs at initial and final time points. **C** Log fold changes of genes after screening with EPIKOL for at least 15 population doublings. **D** Common hits of EPIKOL screens on TNBC (MDA-MB-231, SUM159PT, SUM149PT) and Prostate cancer cell lines (LNCaP, DU145, 22Rv1) identified in *p* < 0.05 cutoff.

To uncover epigenetic modifiers important for cancer cell fitness, we conducted negative selection (drop-out) screens using EPIKOL. Three different triple negative breast cancer (TNBC) cell lines MDA-MB-231, SUM149PT and SUM159PT were screened in addition to non-malignant human mammary epithelium cells (HMLE) [50]. Similarly, prostate cancer (PCa) cell lines LNCaP, DU145 and 22Rv1 were screened along with the normal-like immortalized prostate epithelium cell line RWPE-1. In each screen, Cas9-expressing cell lines were transduced with EPIKOL at a low multiplicity of infection and cultured for 15-16 population doublings (Fig. 2A). To determine relative sgRNA abundance at each timepoint, raw read counts were normalized to reads per million and Log2 transformed (Fig. 2B, Fig. S2A). Model-based Analysis of Genome-wide CRISPR/Cas9 Knockout (MAGeCK) was performed to determine gene-level depletion scores using median normalization and determine the epigenetic modifiers that decrease cell fitness. A number of epigenetic modifiers were found to be significantly depleted in TNBC cell lines MDA-MB-231 (140), SUM149PT (140) and SUM159PT (98). Similar numbers of epigenetic modifiers were depleted in PCa cell lines LNCaP (148), DU145 (181) and 22Rv1 (173) (Fig. 2C, Fig. S2B). Among these, epigenetic modifiers that were previously implicated in breast cancer cell fitness, such as PRMT5 [51], HDAC3 [52], NPM1 [53, 54] were depleted in MDA-MB-231 cells serving as positive controls. Similarly, for prostate cancer, KDM1A [55], BRD4 [56], and PRMT1 [57] were depleted in LNCaP cells as well as AR, FOXA1 and NCOA1 [58, 59], thus serving as positive controls. Well-known cancer survival genes such as PELP1 and PRMT family members were identified as common hits in all the six cancer cell lines screened by EPIKOL (Fig. 2D) [51, 60–63]. These results indicated that our epigenetic-focused screening approach is able to identify genes critical for cancer cell viability. Therefore, we focused on characterizing novel hits from the TNBC screen, which have not been previously linked to TNBC cell viability.

### Effects of novel candidate genes on TNBC cell fitness were validated in dual-color competition assay

**Fig. 3:**
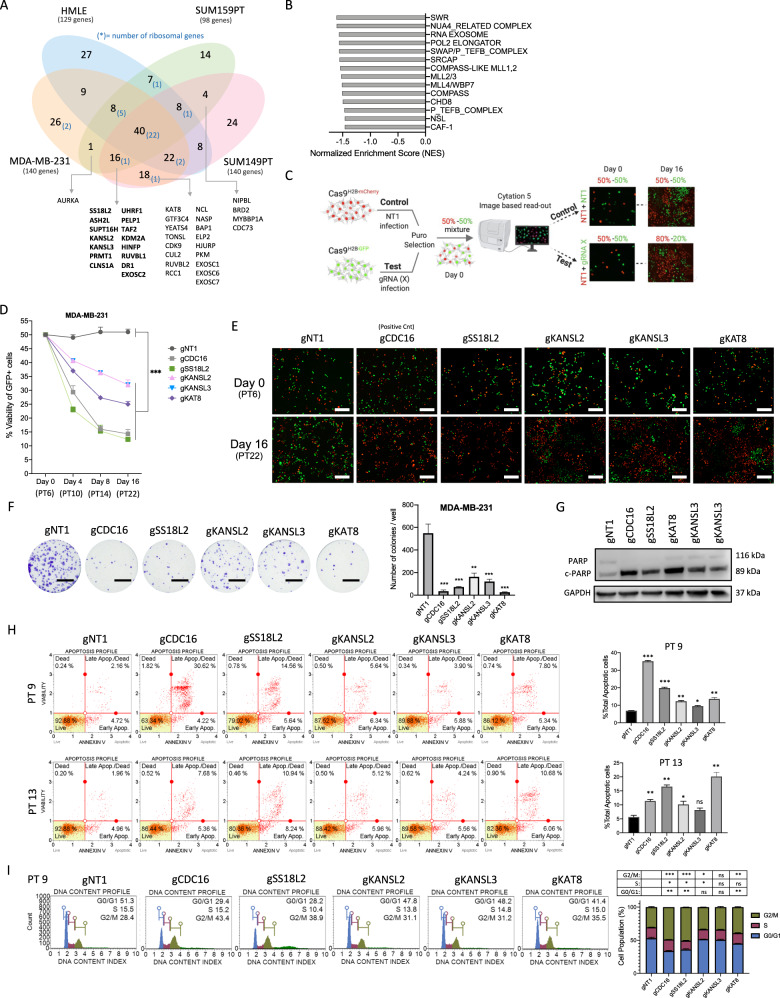
Effects of candidate genes on MDA-MB-231 fitness were validated with functional assays in vitro. **A** Venn diagram showing cell line specific or common genes that are found in *p* < 0.05 cutoff. 15 genes in bold show TNBC specific epigenetic modifiers that were depleted in all three TNBC cell lines. Others are the genes that were commonly depleted in two different TNBC cell lines but not in HMLE. **B** Gene set enrichment analysis with newly curated ‘epigenetic complexes’ gene sets. Normalized enrichment scores demonstrating negative enrichment of epigenetic complexes in MDA-MB-231 cells. **C** Summary of dual-color competition assay for in vitro validation of candidate epigenetic modifiers. NT1: Non-targeting control, CDC16: positive control. **D** Results of dual-color competition assay for selected hits in MDA-MB-231 cells. PT: post-transduction day. **E** Representative images taken with Cytation5 at Day0 and Day16 of competition assay for MDA-MB-231 cells. mCherry+ cells were infected with Non-targeting sgRNA (NT1) as control while eGFP+ cells were infected with sgRNA targeting the gene of interest. Scale bar: 200 µm. **F** Representative images of long-term clonogenic assay for MDA-MB-231 cells infected with sgRNAs against selected hits. Scale Bar: 10 mm.**G** Western Blot analysis of MDA-MB-231 cells after transduction with viruses of indicated sgRNAs at post-transduction day 9. **H** Annexin V & dead cell assay results of selected genes on two different time points and their statistical analysis. **I** Cell cycle analysis of selected genes on post-transduction day9 and its statistical analysis. *P* values determined by two-tailed Student’s *t*-test in comparison to NT1; *P < 0.05, **P < 0.01, ***P < 0.001.

To validate the results of EPIKOL screens, we first identified the genes that were commonly depleted in at least two TNBC cell lines but not significantly depleted in the control HMLE cells (Fig. 3A). From this, 15 genes were found to be essential in all TNBC cell lines including some of the well-known regulators of cancer cell fitness, such as UHRF1 [64], PELP1 [65] and PRMT1 [66]. In addition, we curated epigenetic complex-based gene sets for the genes that are found in EPIKOL to perform Gene Set Enrichment Analysis (GSEA) and expedite the hit selection (Supplementary Table 3). In the MDA-MB-231 screen, several complexes such as MLL and COMPASS-like, RNA exosome, Pol2 elongator and NSL were found to be significantly negatively enriched (Fig. 3B). In total, 40 genes (including several controls) were selected for in vitro validation experiments based on their depletion *p*-values, log fold changes and gene rankings in different screens. 2 sgRNAs per gene were cloned individually into lentiGuide-puro vector and a dual-color competition assay was performed in all TNBC cell lines (Fig. 3C). Cells carrying sgRNAs targeting a hit gene (eGFP+ cells) were outcompeted by the cells carrying non-targeting (NT1) control gRNA (mCherry+ cells) in the cell lines tested (Fig. S3). Of note, depletion ratios varied depending on the cell type; the most significant depletion was observed on MDA-MB-231 followed by SUM149PT and SUM159PT, in line with the depletion ratios observed during EPIKOL screens. The competition assay indicated that shared members of MLL/COMPASS complexes (ASH2L, WDR5, RBBP5) as well as the NuA4 (YEATS4, VPS72) and NSL complex members (KANSL2, KANSL3, KAT8) have strong effects on the fitness of TNBC cell lines. Collectively, these findings show that EPIKOL screens identified novel epigenetic modifiers that regulate TNBC cell fitness.

### Knockout of individual epigenetic modifiers caused growth defects in TNBC cell line MDA-MB-231

To further delineate the effects of novel epigenetic modifiers that regulate cell fitness, four of the TNBC specific genes (SS18L2, KANSL2, KANSL3 and KAT8) were selected based on their strong depletion scores in MDA-MB-231 (Fig. 2C). Three of these genes belong to the same complex, namely the non-specific lethal (NSL) complex. KANSL2 and KANSL3 are structural components of NSL complex together with KANSL1. KAT8 (*MOF, MYST1*) is the catalytic member of the complex and acetylates histone lysine residues [67]. SS18L2 is the homolog of the *SS18* gene, which is associated with chromosomal translocation characteristics of synovial sarcoma. However, the exact role of SS18L2 in synovial sarcoma or any other cancer is not known [68]. NSL complex members (KANSL2, KANSL3, KAT8) and SS18L2 showed a strong TNBC-specific effect in EPIKOL screens. In competition assays, cells carrying sgRNAs targeting all four hit genes were significantly depleted in MDA-MB-231 cells over 16 days (Fig. 3D, E). Long-term colony formation assays demonstrated that knockouts of all selected genes exert strong fitness defects (Fig. 3F). Suppression of these genes led to 65–75% fewer number of colonies compared to control conditions with the SS18L2 and KAT8 depletion phenotype reaching to the level observed with the depletion of positive control CDC16. Interestingly, knockout of close homologs of SS18L2 (SS18 and SS18L1) did not alter cell fitness as there was no change in the colony formation assay (Fig. S4A). Taken together, these results show that knocking out either SS18L2 or members of NSL complex have a profound effect on TNBC cell fitness.

### Knockout of epigenetic modifiers induced apoptosis in MDA-MB-231 cells

To identify the mechanism through which cancer cell fitness is reduced, we first investigated whether knockout of the hit genes result in apoptosis. Extensive cleavage of PARP was observed with all four gene knockouts, indicating induction of apoptosis (Fig. 3G). Similarly, Annexin V & Dead cell staining showed significantly more cells in early- and late-apoptotic states upon knockout when compared to control cells (Fig. 3H) at two different timepoints. 9 days post sgRNA transduction, knockout of SS18L2 induced significant level of apoptosis, in line with the effect observed in the first four days of competition assays (Fig. 3D). On the other hand, the effect of knocking out NSL complex members, especially of KAT8, were more pronounced at PT13 (13 days post sgRNA transduction). We also observed a reduced number of cells in the G0/G1 and S phases of cell cycle and accumulation at G2/M phase upon SS18L2 and KAT8 knockout (Fig. 3I). This indicates that knockout of these genes may also result in mitotic arrest. Collectively, these findings suggest that four candidate genes are essential to TNBC cells, which might be exploited for therapeutic purposes. These proof-of-principle experiments demonstrate that our focused epigenome-wide CRISPR library, EPIKOL, is an easy-to-use functional genomics tool that enables the identification of epigenetic modifiers important for cancer cell fitness.

### In vivo EPIKOL screen identified SS18L2 essential for TNBC tumor growth

**Fig. 4:**
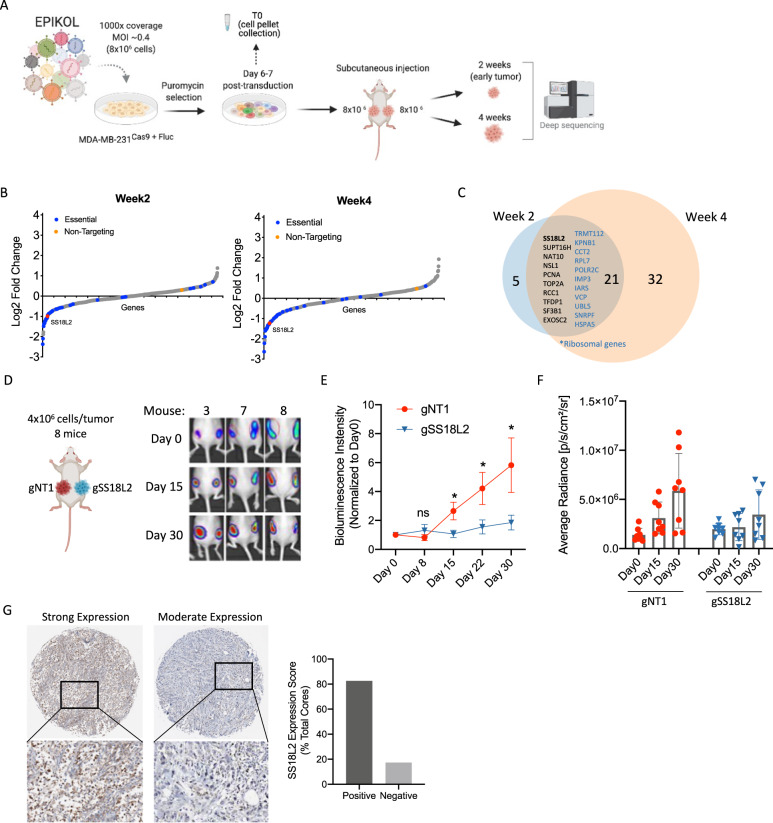
In vivo EPIKOL screen identified SS18L2 essential for TNBC cell survival. **A** Summary of in vivo screening procedure. **B** Log fold changes of genes after in vivo screening with EPIKOL for 2- and 4-weeks. **C** Common hits of EPIKOL screens from week 2 and 4 tumors identified in *p* < 0.05 cutoff. **D** Individual validation of effects of SS18L2 knockout tumors when compared to Non-targeting gRNA containing tumors injected subcutaneously to the flank regions. Representative images show day0-15-30 bioluminescence measurements of three mice (*n* = 8 per group). **E** Bioluminescence of tumors containing SS18L2 sgRNA normalized to control group containing non-targeting gRNA (*n* = 8/group) for 30 days period. **F** Average radiance of all tumors on day 0, day 15 and day 30. **G** Representative core images from breast tissue microarrays stained with anti-SS18L2 antibody and percentage of SS18L2-positive cores (*n* = 132 cores). Digital scores over 0.1 was considered positive. *P* values determined by two-tailed Student’s *t*-test in comparison to NT1; *P < 0.05.

To assess the performance of EPIKOL in vivo, we performed an in vivo screen by using Firefly Luciferase (Fluc) expressing MDA-MB-231-Cas9 cell line. Following transduction and puromycin selection, cells were subcutaneously injected into Nude mice, and tumors were collected at weeks 2 and 4 post-implantation (Fig. 4A). Gene-level waterfall plots demonstrated the depletion of essential genes and stationary behavior of non-targeting controls during the screen (Fig. 4B). Overall depletion ratios and numbers of significantly depleted essential genes were increased in week 4 tumors compared to week 2 tumors. In addition to the positive control essential genes, 10 epigenetic modifiers were identified as commonly depleted in both timepoints. Notably, SS18L2 was among this set of significantly depleted genes (Fig. 4B, C). To validate the effects of SS18L2 knockout in vivo, we transduced MDA-MB-231-Cas9-Fluc cells with either NT1 or SS18L2 targeting sgRNAs and injected them subcutaneously into Nude mice. Normalized bioluminescence intensities of tumors showed that the tumors carrying SS18L2 sgRNA did not grow in vivo when compared to NT1-tumors (Fig. 4D–F). To assess if SSL182 is expressed in human breast cancers, we performed immunohistochemistry on a breast tissue microarray that mostly include TNBC samples and observed that 80% of TNBC cores had moderate to strong SS81L2 expression (109 out of 132 cores) (Fig. 4G, Fig. S5, Supplementary Table 4).

### SS18L2 is required for G2/M transition in TNBC cells

**Fig. 5:**
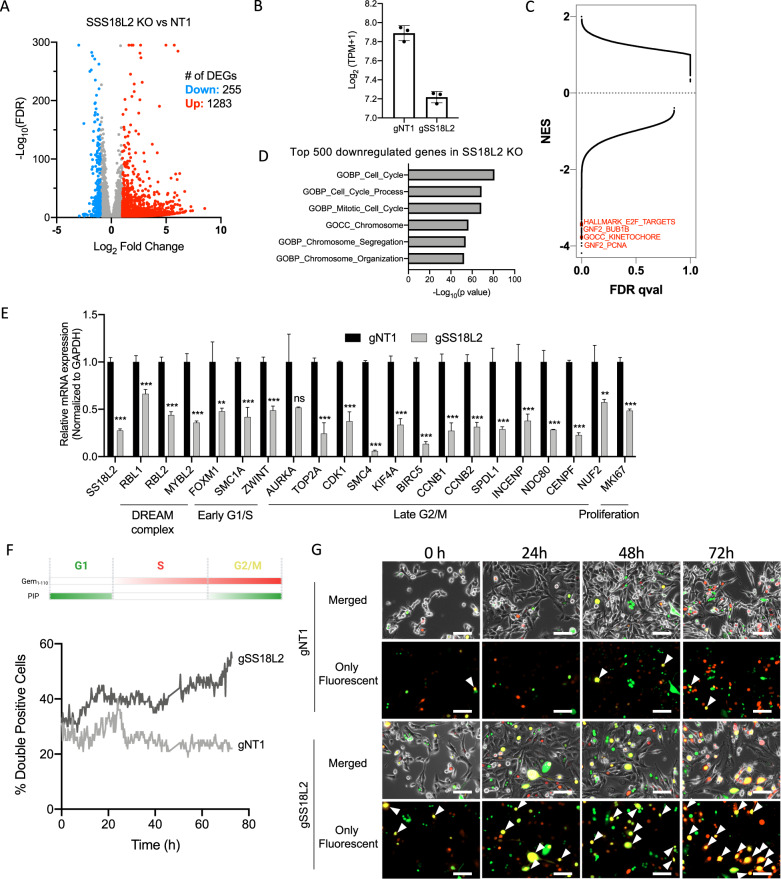
Knockout of SS18L2 causes G2/M cell cycle arrest. **A** Volcano plot showing differentially expressed genes (DEGs) in SS18L2-KO MDA-MB-231 cells when compared to control on the 6^th^ day of transduction **B** Transcript per million (TPM) counts of SS18L2 in SS18L2-KO and control (NT1) samples. **C** Normalized enrichment score and FDR-qval results of gene set enrichment analysis (GSEA) of all genes for all gene sets available from MSigDB v7.5. Some of the negatively enriched pathways related to cell cycle were highlighted. **D** Top 6 biological processes enriched in downregulated genes upon SS18L2 knockout. *P* values were calculated by hypergeometric test. Top 500 most downregulated genes were used during the analysis. **E** Quantitative real-time pcr analysis of downregulated genes upon SS18L2 knockout. **F** Schematic for PIP-FUCCI cell cycle analysis and percentages of cells that express both mVenus and mCherry as an indicative of cells in G2/M phase. Cells were imaged starting from post-transduction day 4 to day 6 with 15-min intervals from four independent areas of the wells. **G** Representative images of PIP-FUCCI experiment taken with Cytation5. Arrow heads indicate double fluorescent yellow cells. Scale bar: 100 µm. *P* values determined by two-tailed Student’s *t*-test in comparison to NT1; *P < 0.05, **P < 0.01, ***P < 0.001.

To gain a mechanistic understanding for the essential role of SS18L2 in TNBC cells, we analyzed the transcriptomic changes caused by the knockout of SS18L2. We performed RNA sequencing comparing MDA-MB-231-Cas9 cells expressing control and SS18L2 sgRNAs. SS18L2 knockout resulted in 1283 upregulated and 255 downregulated genes (Fig. 5A). Average expression of SS18L2 in all replicates were significantly downregulated upon sgRNA transduction when compared to control (Fig. 5B). GSEA revealed a number of negatively enriched pathways related to cell cycle with significant normalized enrichment scores (Fig. 5C). Similarly, biological processes analysis from the Molecular Signature Database (MsigDB) revealed that downregulated genes upon SS18L2 knockout significantly overlapped with cell cycle, mitosis, chromosome organization and segregation related gene sets (Fig. 5D). On the other hand, upregulated genes did not significantly overlap with any particular pathway, indicating that the major effect of SS18L2 knockout is downregulation of a specific group of cell cycle-related genes. We confirmed the differential expression of cell cycle-related genes belonging to DREAM complex, early G1/S, and late G2/M phase by qPCR (Fig. 5E). To validate the effect of downregulated genes on cell cycle phase transitions, we conducted a time-lapse PIP-FUCCI fluorescence imaging for 72 h [49]. SS18L2 knockouts had significantly lower numbers of cells when compared to control cells at the end of 72 h, indicating a decrease in proliferation rate (Fig. 5G). Morphologically, SS18L2 knockout cells were larger in size resembling senescent cells. More importantly, the number of mVenus and mCherry double-positive cells, indicative of G2/M phase, was significantly increased upon SS18L2 but not SS18 or SS18L1 knockouts (Fig. 5F, Fig. S4B). This finding suggests that upon SS18L2 knockout, cells can enter the cell cycle but cannot complete mitosis and become arrested. Collectively, these results show that EPIKOL is a robust tool to identify essential epigenetic modifiers both in vitro and in vivo and that SS18L2 plays a critical role in maintenance of TNBC cell fitness in part by regulating cell cycle progression.

## Discussion

In this study, we present a focused epigenetic knockout library (EPIKOL) that can be utilized to investigate chromatin-based vulnerabilities in different biological contexts. We performed eight in vitro screens in two cancer types and identified novel chromatin modifiers that regulate prostate and triple-negative breast cancer cell fitness. In contrast to most currently available epigenome-focused libraries, which only target chromatin modifiers such as writers, readers, and erasers [40–42], EPIKOL targets a wider range of genes encoding chromatin complex cofactors and structural components [44]. Thus, its use will likely lead to a broader understanding of the functions of these complexes as a whole.

Availability of EPIKOL in LentiCRISPRv2 backbone might expedite the screening process by eliminating the need for prior Cas9 introduction especially in patient-derived xenograft models and primary cell lines, in which the culturing time of the material is limited. In such cases, smaller library size will also reduce the amount of initial material required to maintain the complexity. Another advantage of EPIKOL is the presence of sgRNAs targeting context-specific control genes from different families such as nuclear receptors, transporters and EMT-related proteins. For example, Androgen Receptor (AR) targeting sgRNAs were significantly depleted in AR-dependent prostate cancer cell lines LNCaP and 22Rv1 while no change was observed in AR-negative cell line DU145 and TNBC cell lines suggesting that EPIKOL can distinguish tissue or cell line specific hits.

From drop-out screens in multiple cell lines, we identified novel epigenetic modifiers for cancer cell fitness as well as the previously studied ones such as PRMT5, HDAC3, FOXA1 and LSD1 [51, 52, 59, 60]. Comparisons between six different cancer cell lines revealed 25 epigenetic modifiers commonly depleted in all cell lines. Among them, several genes belong to the same complex such as *PRMT* family, exosome complex and MLL complexes, highlighting the role of these epigenetic complexes as pan-cancer essential epigenetic modifiers. Some of these were previously identified as common essential genes in Cancer Dependency Map (DepMap) based on their significant depletion in almost 750 different cancer cell lines [69]. Notably, we identified *ASH2L* as a common essential gene in both cancer types through EPIKOL screens, but it was not classified as a common essential gene in previously performed screens. EPIKOL screen can therefore identify pan-cancer epigenetic vulnerabilities as well as cell line and cancer-specific ones. To test the performance of EPIKOL in vivo, we performed a screen using MDA-MB-231-Cas9-Fluc cells at two different timepoints. As expected, overall depletion scores and number of depleted positive control genes increased from week 2 to week 4. 9 out of 10 commonly depleted genes from both timepoints of the in vivo screen were shared with the TNBC in vitro screens. TFDP1 was identified as an in vivo specific cell fitness gene. These results indicate that EPIKOL efficiently works in vivo and can recapitulate the findings of in vitro screens.

We identified a group of epigenetic modifiers that belong to NSL complex as TNBC-specific cell fitness genes. Core members KANSL2 and KANSL3 were depleted in three different TNBC cell lines but they did not show significant effects in HMLE cells. Importantly, KAT8, the catalytic subunit of the NSL complex, was also a common hit in MDA-MB-231 and SUM149PT cell lines. We showed that TNBC cells were dependent on NSL complex using several functional assays. NSL complex was thought to regulate H4K16Ac which is necessary for chromatin relaxation and present at active enhancers and promoters [70, 71]. However, the role of the NSL complex in cancers has been controversial [72–76]. A recent study showed that KAT8 mainly regulates H4K5Ac and H5K8Ac as a member of NSL complex, while it regulates H4K16Ac as a member of MSL complex. Activity of NSL complex for H4K5Ac and H4K8Ac is critical for cancer cell survival, while the activity of MSL complex and H4K16Ac is not. They also suggested that complete loss of KAT8 might be more detrimental for KAT8-low tumors as this would completely destroy the activity of NSL complex [67]. To the best of our knowledge, there is no study showing how KAT8 and NSL complex regulate TNBC cell fitness and survival. Here, we showed that KAT8 together with the two structural components of NSL complex (KANSL2 and KANSL3), decrease TNBC cell fitness and induce apoptosis while neither of MSL complex members were identified as essential. Collectively, these results highlight the effect of NSL complex as a regulator of cell fitness in TNBC.

In addition to the NSL complex, we observed SS18L2 to be significantly depleted in all TNBC EPIKOL screens. Competition assays confirmed the strong effect of SS18L2 knockout on TNBC cell survival. *SS18L2* is a homolog of *SS18*, which is found in ATP-dependent chromatin remodeling complex BAF (SWI/SNF) and an uncharacterized gene [68]. Together with BAF47, SS18 regulates normal expression patterns from enhancers and promoters; however malignant gene translocation (SS18-SSX) in synovial sarcoma evicts BAF47 from the complex and new fusion oncoprotein activates bivalent genes [77]. The role of SS18L2 is not known in cancers and there is no study showing the effect of this gene in TNBC. Here, for the first time, we showed that knockout of SS18L2 decreased TNBC cell survival and concomitantly induced G2/M arrest and apoptosis. Knockout of SS18L2’s close homologs SS18 and SS18L1 did not exert any effects on TNBC cell fitness, suggesting that SS18L2 might have unique properties. Notably, one of the most significantly depleted genes in in vivo screen was *SS18L2*. Knockout of SS18L2 markedly impaired tumor growth confirming that SS18L2 is required for TNBC cells in vivo. Transcriptomic analyses showed that SS18L2 regulates cell cycle-related pathways and genes that function during the late G2/M phase. For example, the DREAM complex activates late G2/M genes through its interactions with *MYBL2* and *FOXM1*, both of which are downregulated upon SS18L2 suppression [78, 79]. In light of these data, we hypothesized that SS18L2 might be an upstream regulator of the late G2/M genes. Functional assays confirmed that SS18L2 knockout cells become arrested in G2/M. Although SS18L2 is an uncharacterized gene, a recent study performed proximity labeling for SS18L2 in HEK293T cells and identified that it binds to BAF complex members [80]. In the future, it will be valuable to unravel interacting partners of SS18L2 in TNBC to better understand how SS18L2 regulates cell cycle and cell fitness. Interestingly, previously published epigenome-wide libraries did not include KANSL2, KANSL3 and SS18L2 targeting sgRNAs, and therefore failed to identify strong effects of these genes on cell survival [40–42].

In conclusion, we generated and validated a focused epigenetic sgRNA library that enables identification of critical epigenetic modifiers both in vitro and in vivo. Epigenetic modifying enzymes are promising therapeutic targets as they regulate numerous critical cellular responses including cell growth, metastasis, apoptosis and others. The relatively small library size both allows for loss-of-function screens where cell numbers are limited, and also provides a focused perspective for hit prioritization. EPIKOL is therefore a robust functional genomics platform to interrogate chromatin modifiers and can guide the discovery of cell-type specific epigenetic vulnerabilities of cancers.

## Supplementary information

Co-author agreement document

Supplementary information

FULL LENGTH WESTERN BLOT IMAGES

Supplementary Table 1

Supplementary Table 2

Supplementary Table 3

Supplementary Table 4

Reproducibility checklist file

## Data Availability

EPIKOL screen and RNA sequencing data are deposited to the NCBI GEO database with the accession number GSE173892.
