# Supplementary material for: EPIKOL, a chromatin-focused CRISPR/Cas9-based screening platform, to identify cancer-specific epigenetic vulnerabilities
Source: Cell Death Dis. 2022 Aug 16;13(8):710. doi: 10.1038/s41419-022-05146-4 (PMC9381743; doi:10.1038/s41419-022-05146-4)

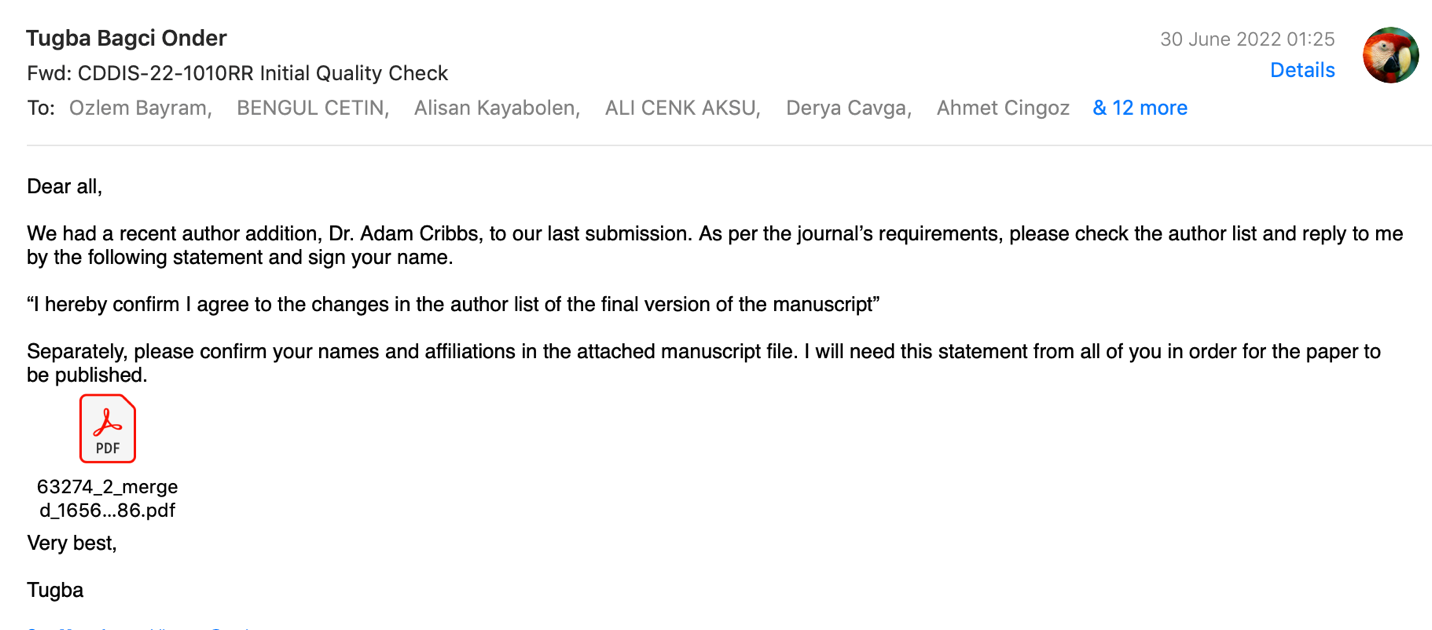


Reply from Ozlem Yedier-Bayram:


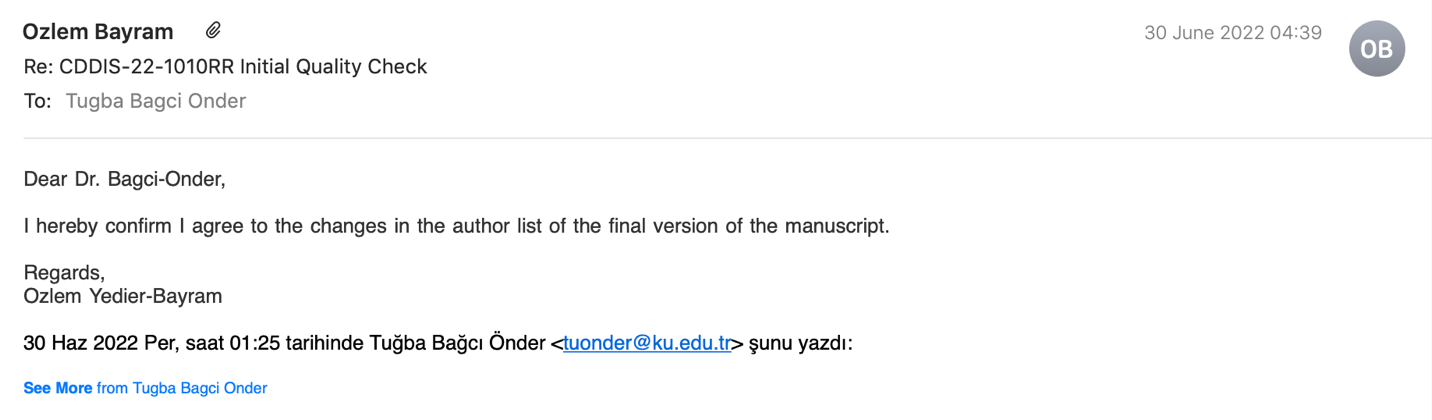


Reply from Bengül Gökbayrak:


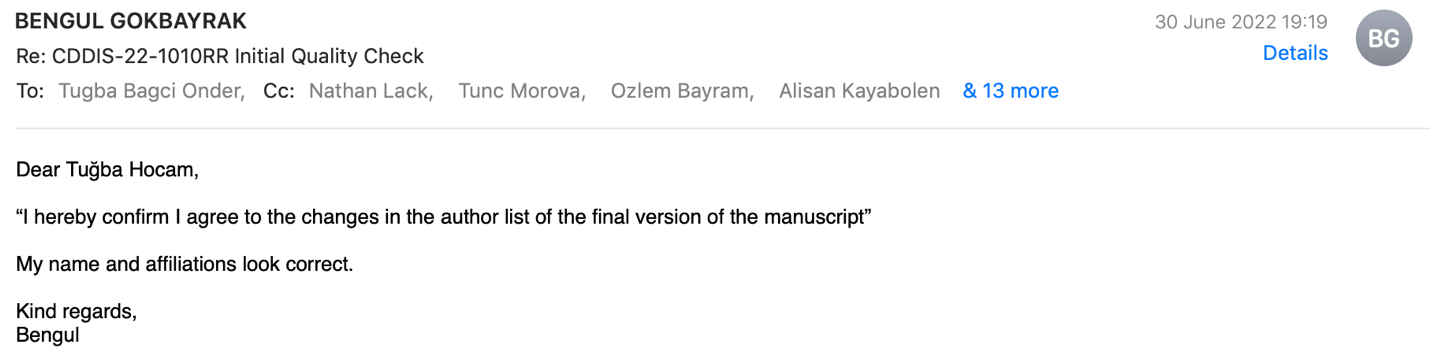


Reply from Alişan Kayabölen:


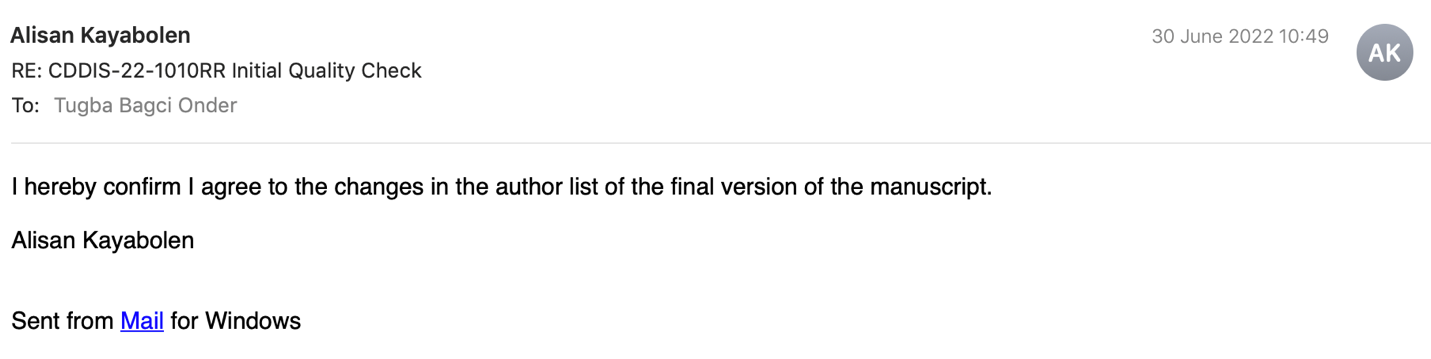


Reply from Ali Cenk Aksu:


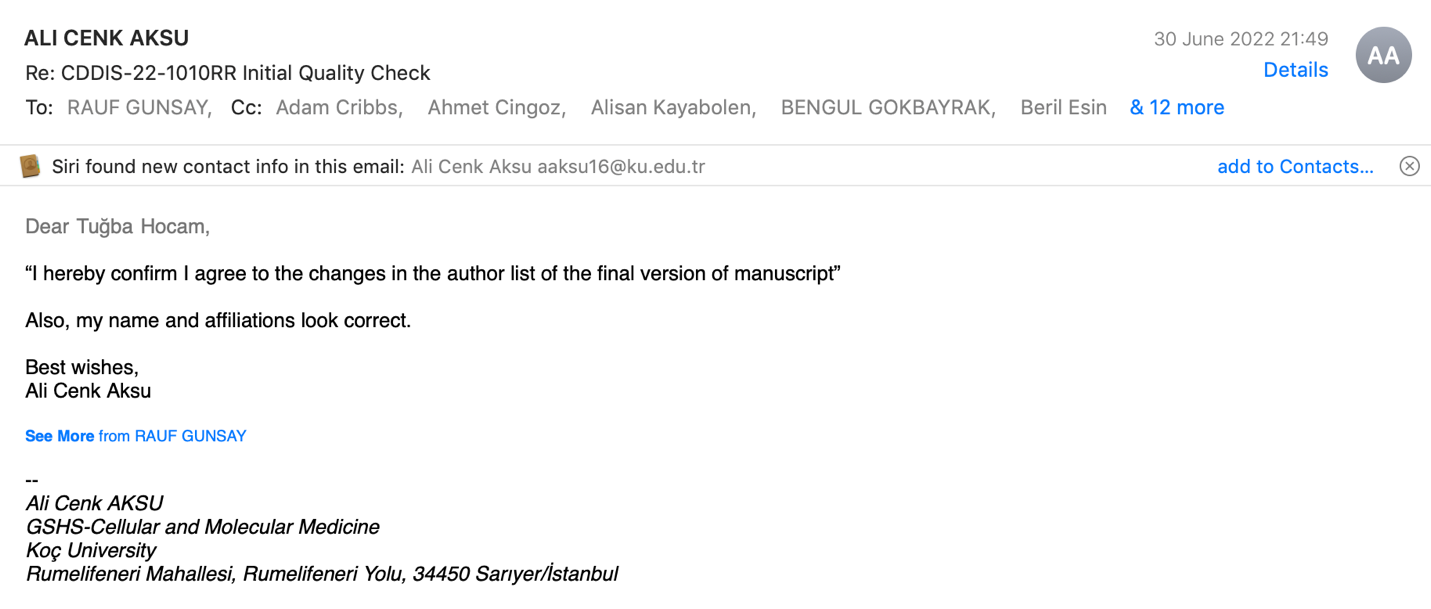


Reply from Ayse Derya Cavga:


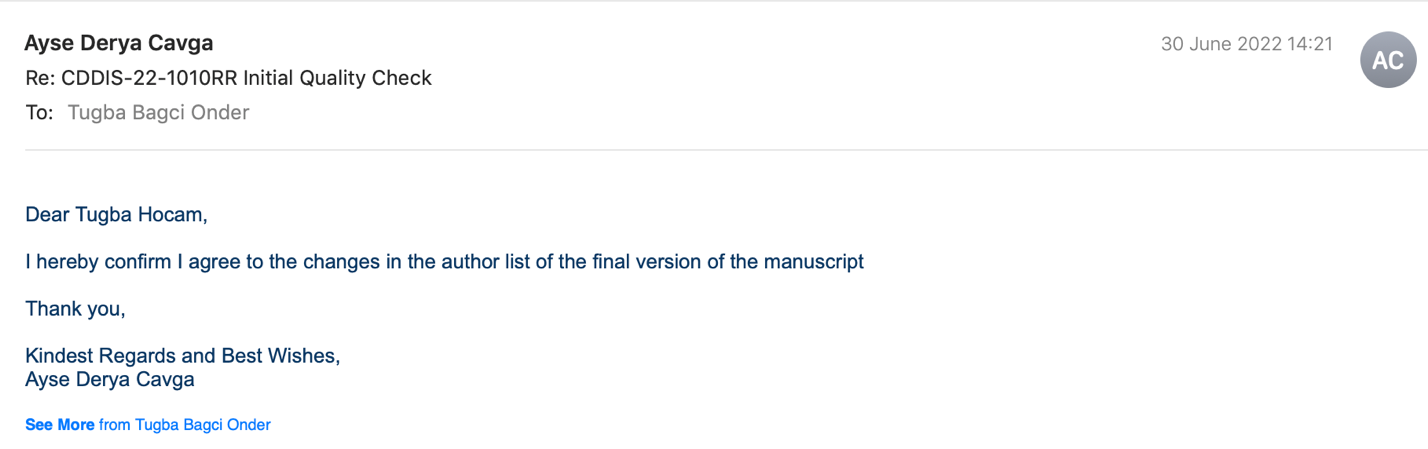


Reply from Ahmet Cingoz:


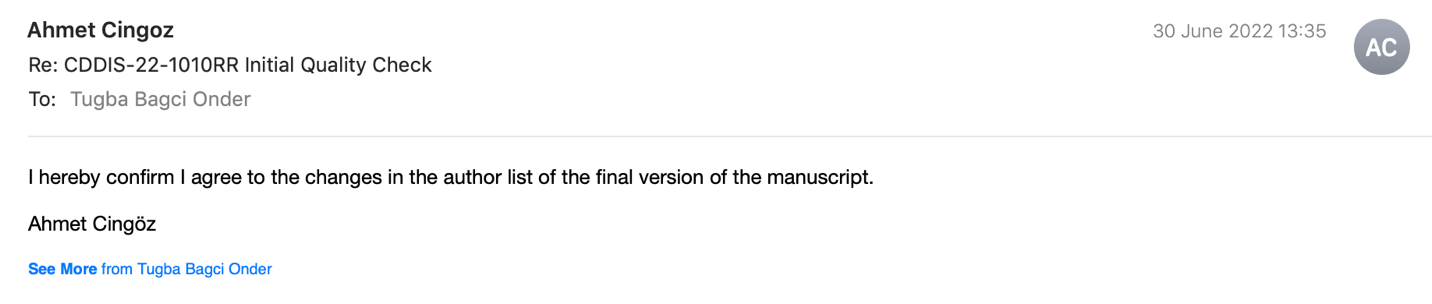


Reply from Ezgi Yagmur Kala:


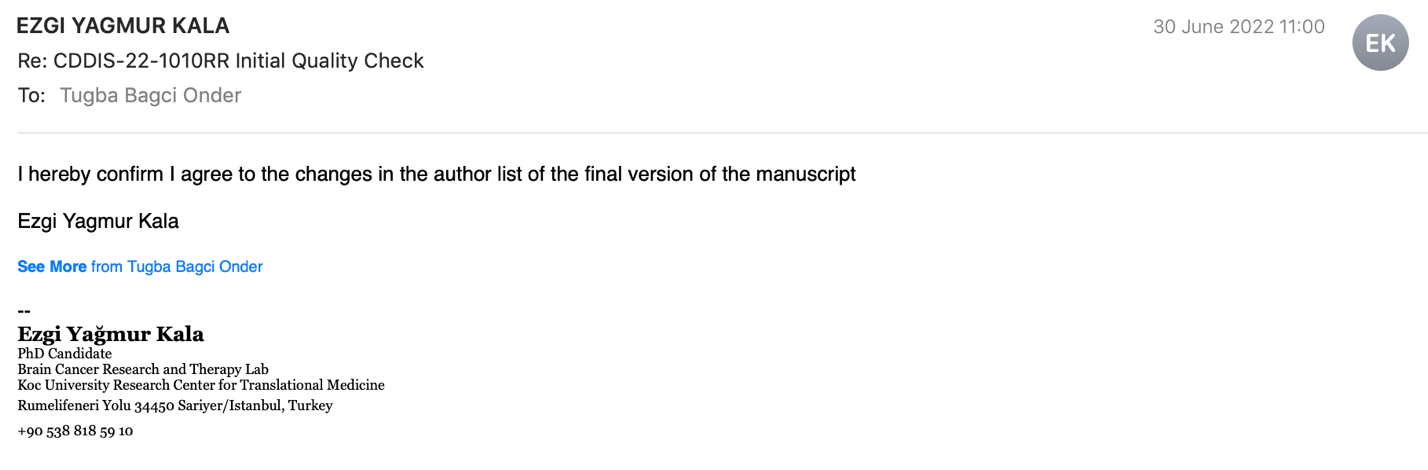


Reply from Goktug Karabiyik:


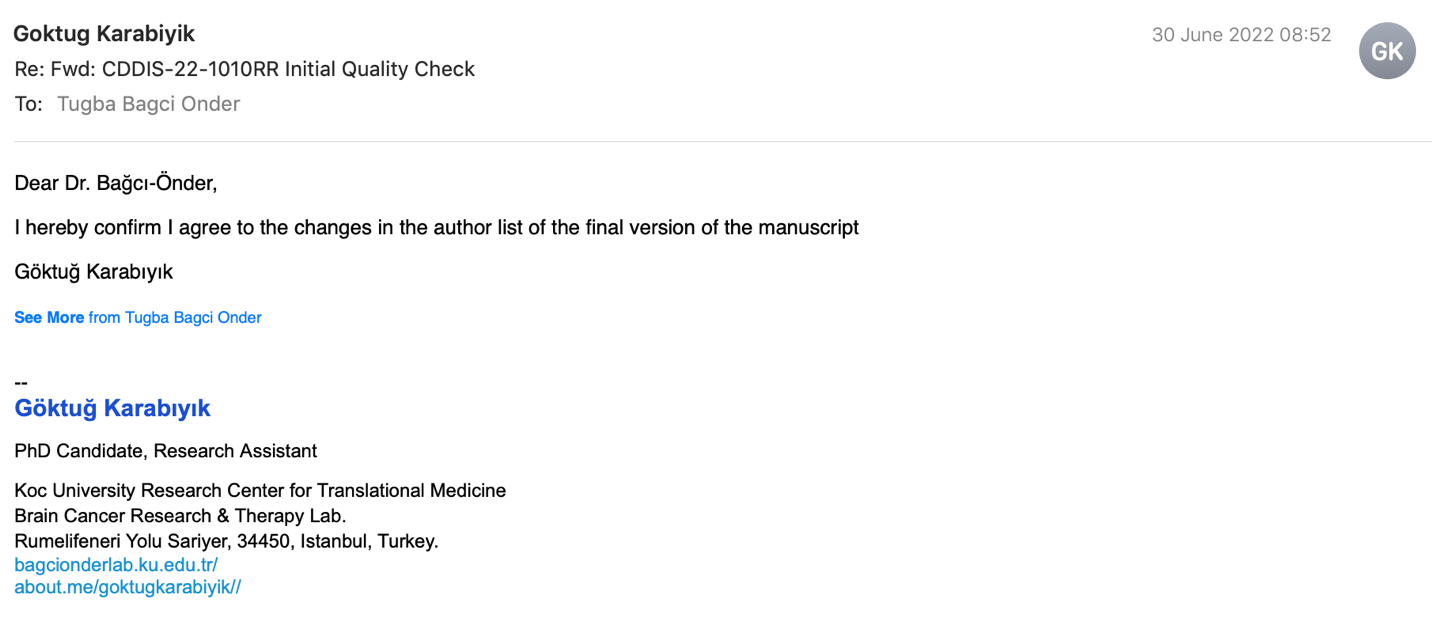


Reply from Rauf Gunsay:


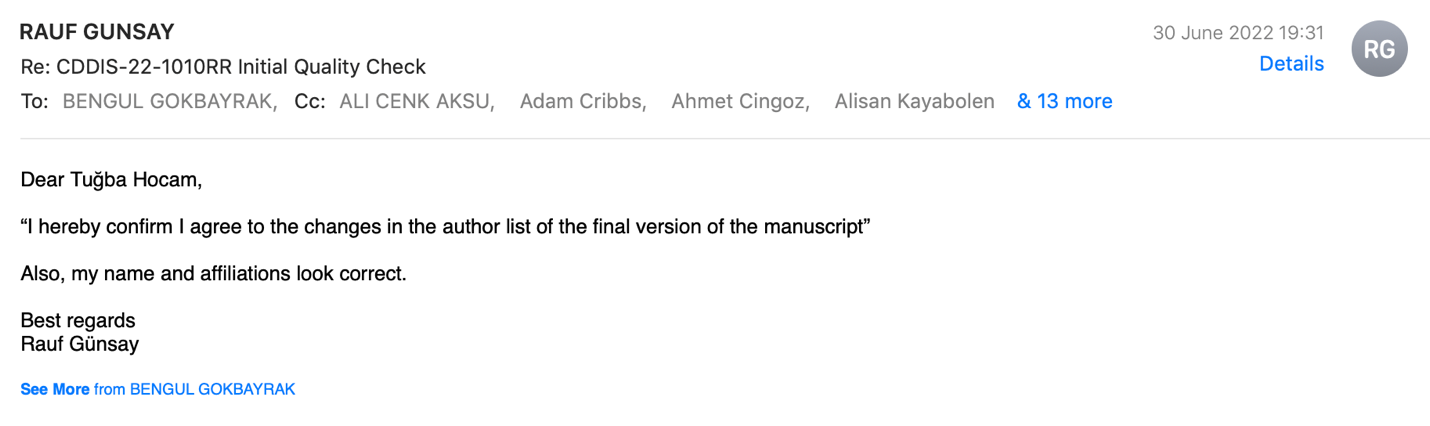


Reply from Beril Esin:


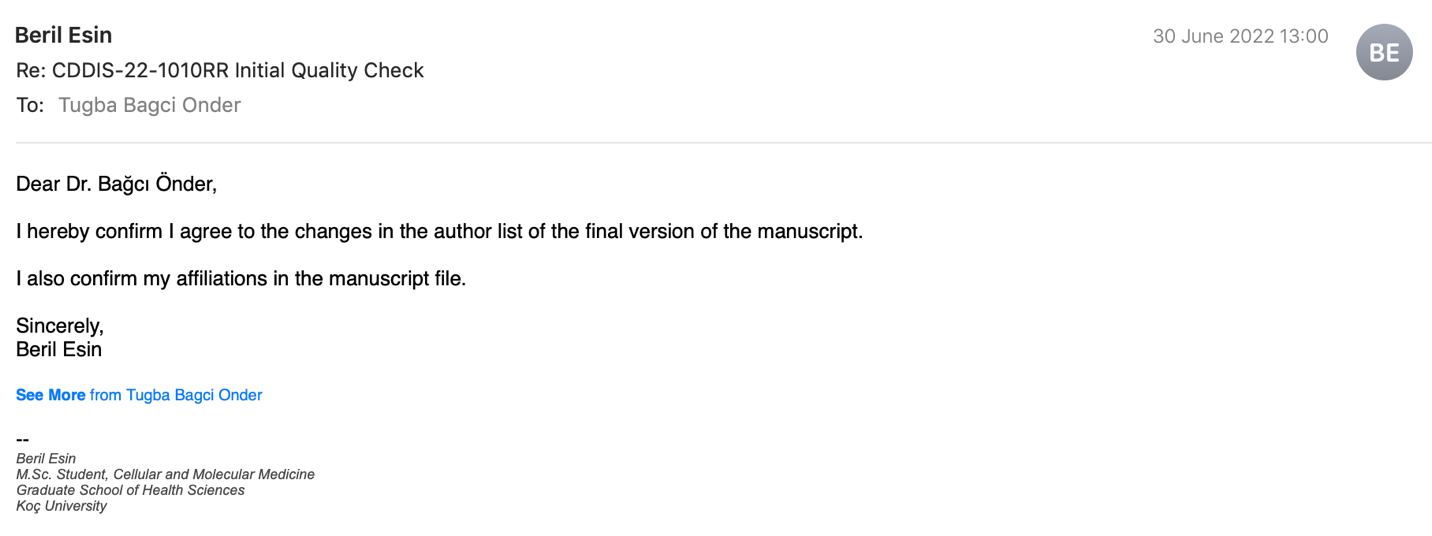


Reply from Tunc Morova:


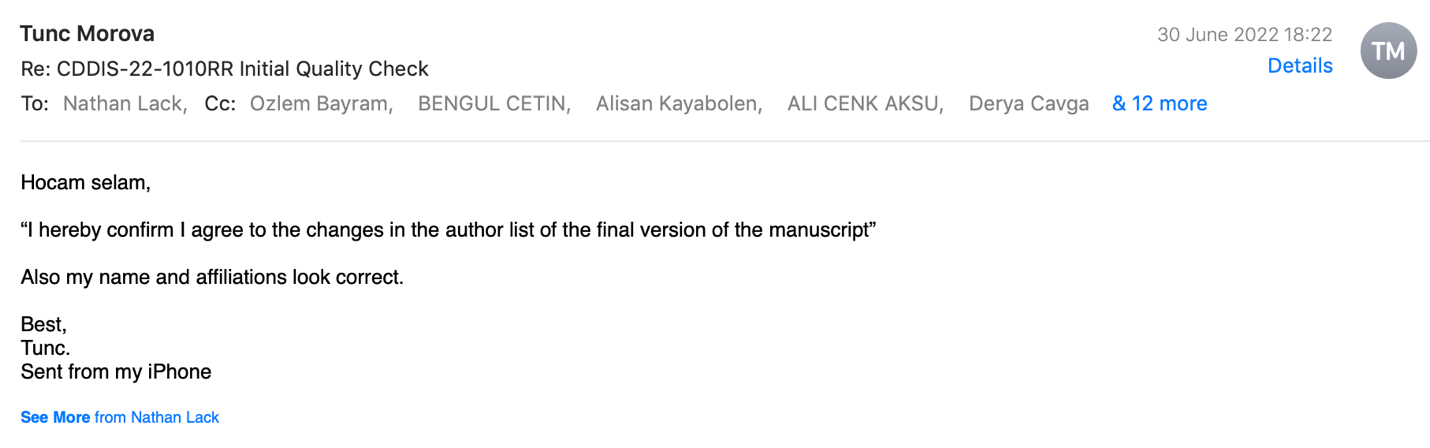


Reply from Firat Uyulur:


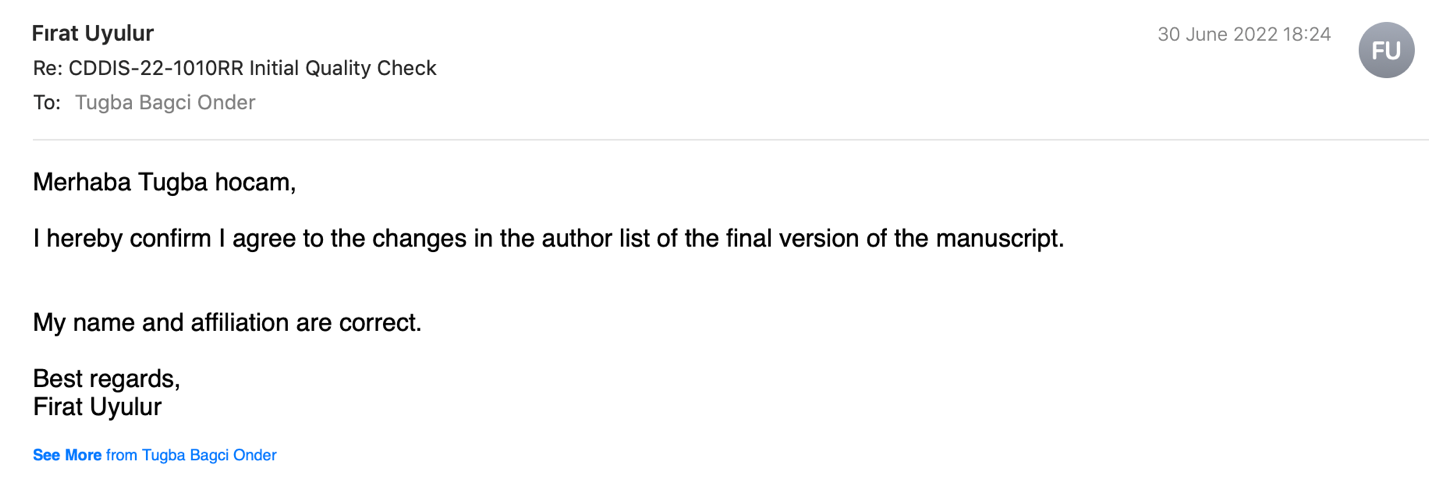


Reply from Hamzah Syed:


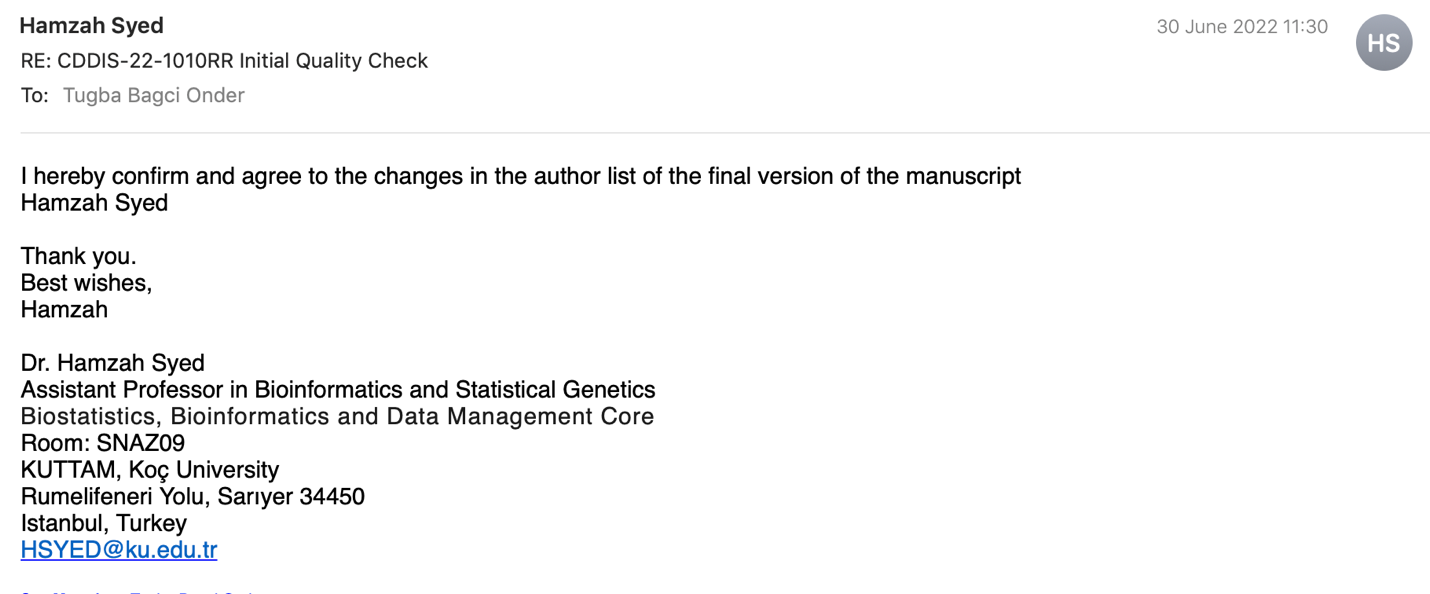


Reply from Martin Philpott:


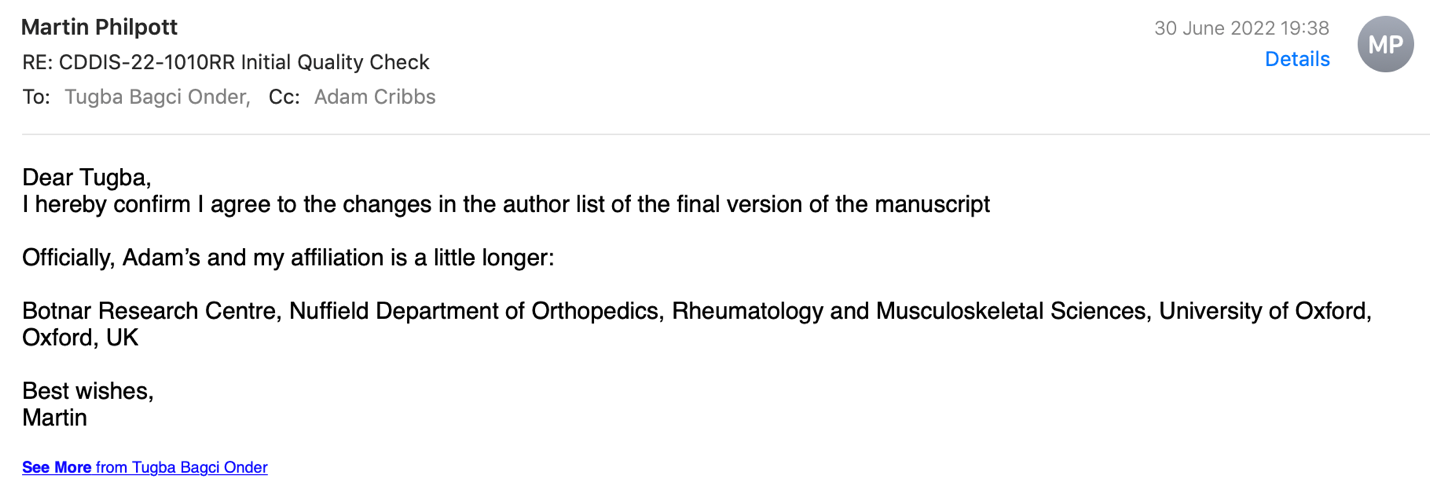


Reply from Adam Cribbs:


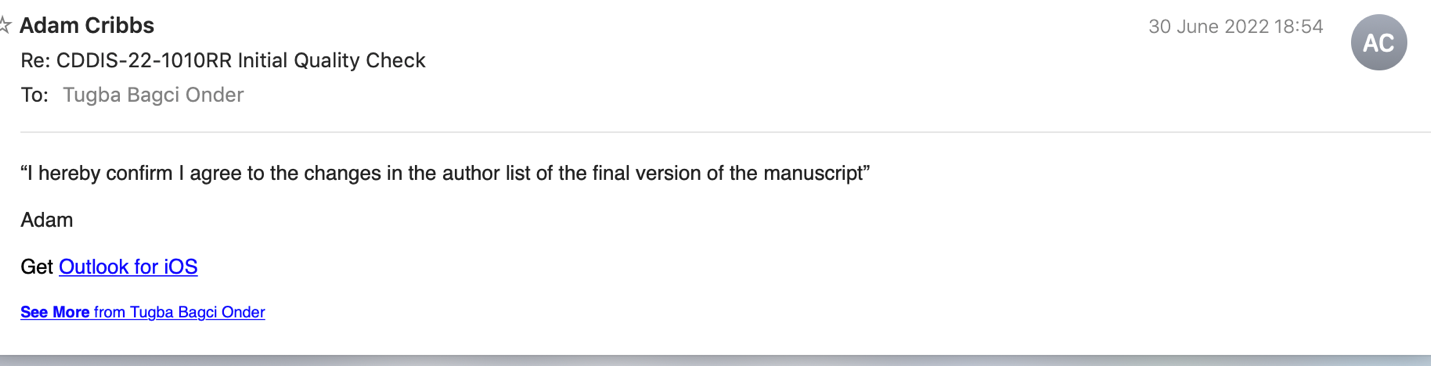


Reply from Sonia HY Kung:


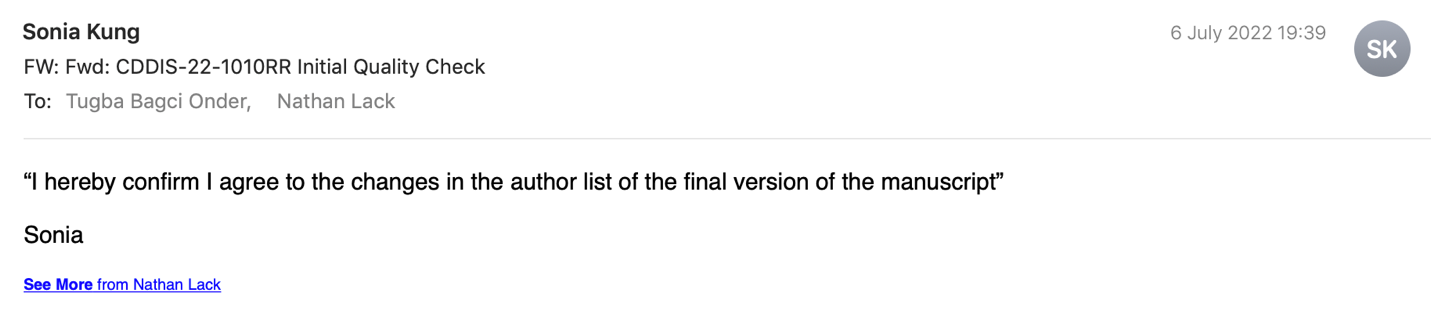


Reply from Nathan A Lack:


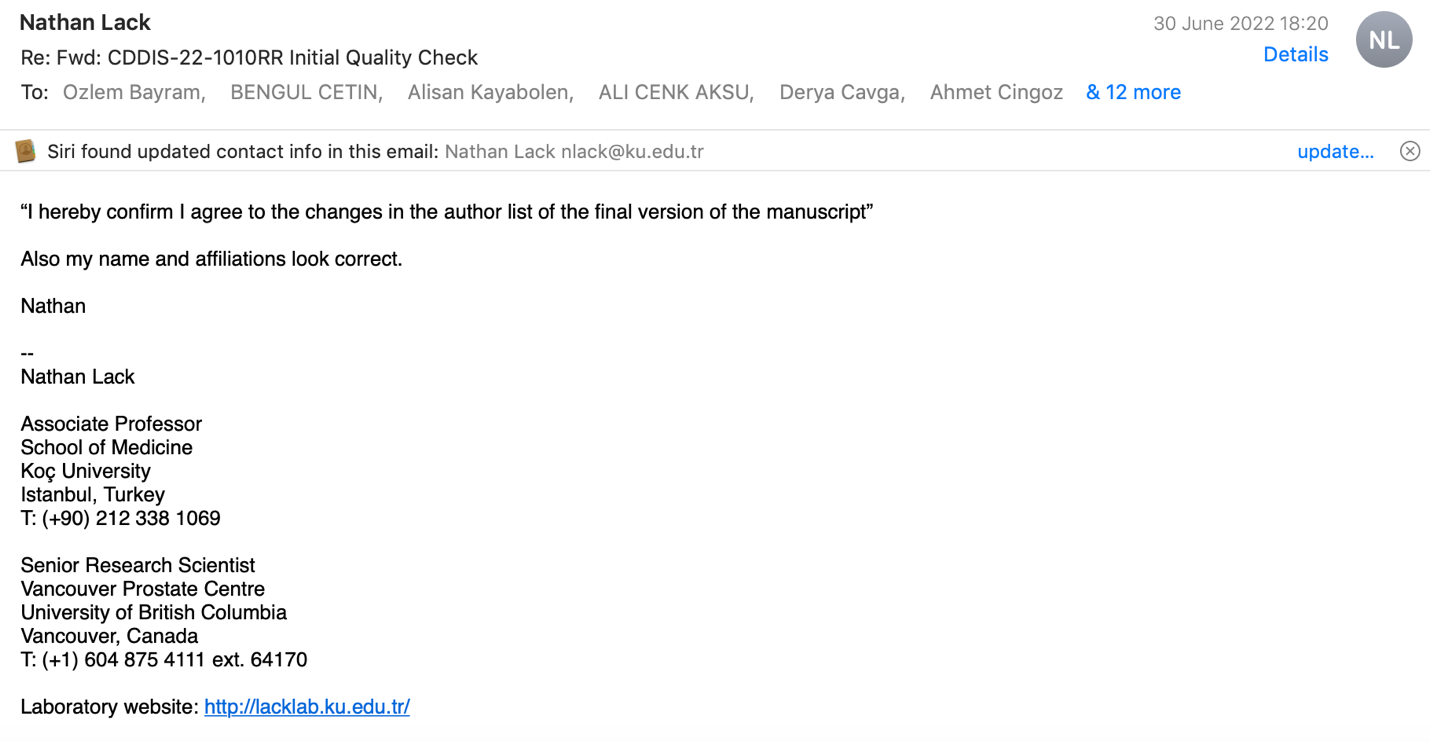


Reply from Tamer T Onder:


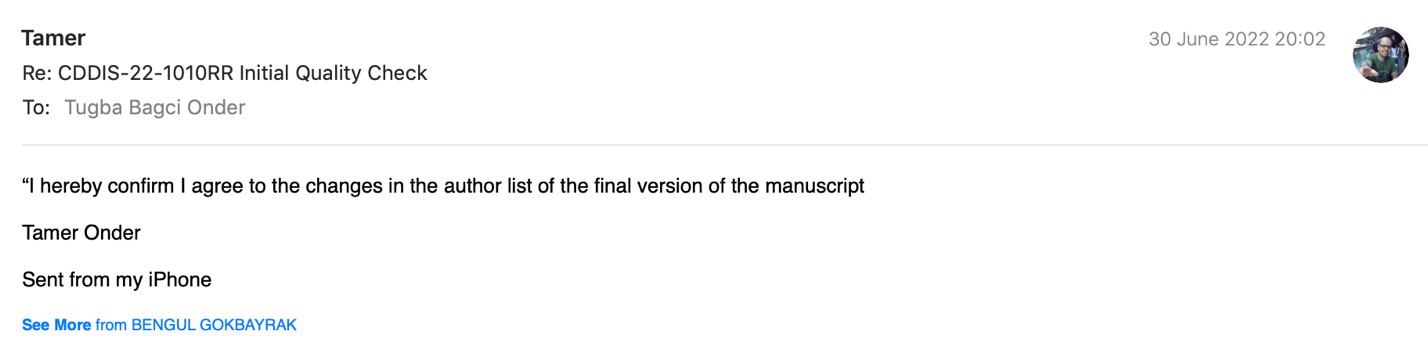

Supplement: Supplementary file 1 — Co-author agreement document [file 41419_2022_5146_MOESM1_ESM.docx]
