# Supplementary material for: EPIKOL, a chromatin-focused CRISPR/Cas9-based screening platform, to identify cancer-specific epigenetic vulnerabilities
Source: Cell Death Dis. 2022 Aug 16;13(8):710. doi: 10.1038/s41419-022-05146-4 (PMC9381743; doi:10.1038/s41419-022-05146-4)
Supplement: Supplementary file 2 — Supplementary information [file 41419_2022_5146_MOESM2_ESM.pdf]

SUPPLEMENTARY INFORMATION

1- SUPPLEMENTARY FIGURES

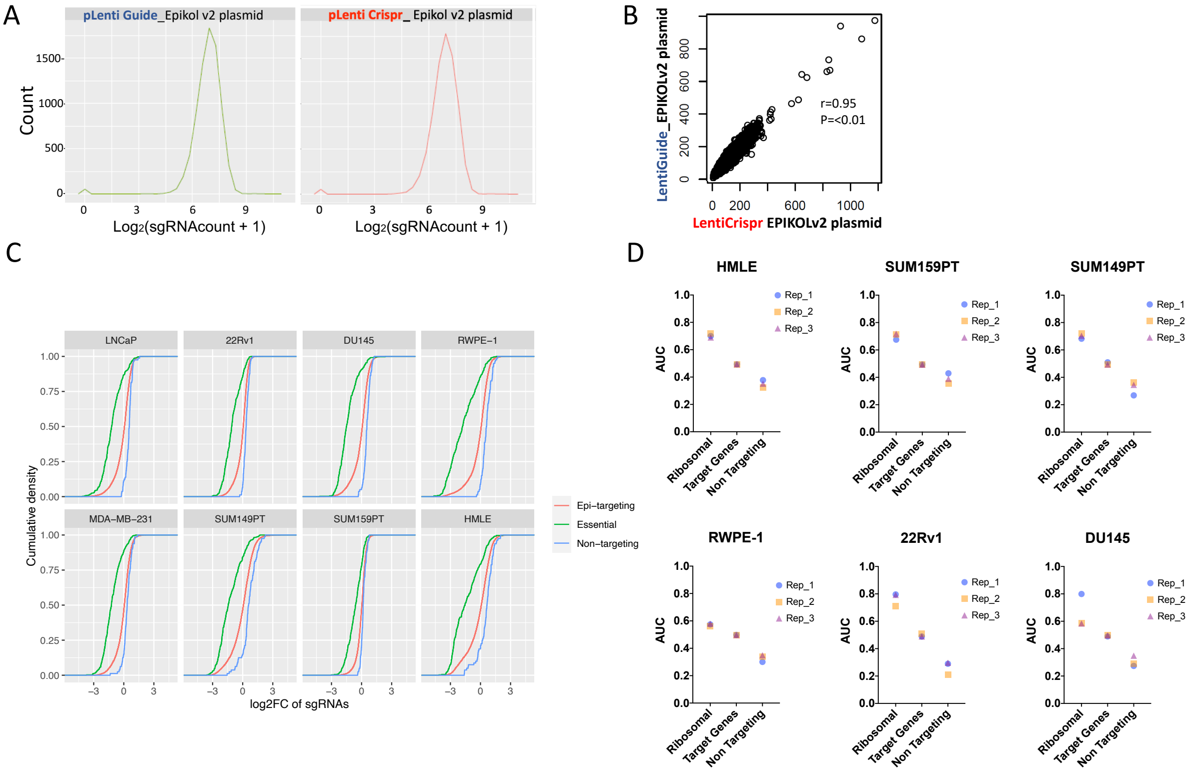

Supplementary Figure 1. Quality Check of EPIKOL in plasmid and transduced cell level ***A.*** Density and correlation plots for EPIKOL in LentiGuide or LentiCRISPRv2 backbone. ***B***. Correlation analysis of EPIKOL amplified from two different plasmids. ***C.*** Cumulative density plots showing differential depletion of essential genes during screen when compared to epi-targeting genes or non-targeting controls ***D.*** Area Under the Curve (AUC) calculations for EPIKOL screens in all cell lines.

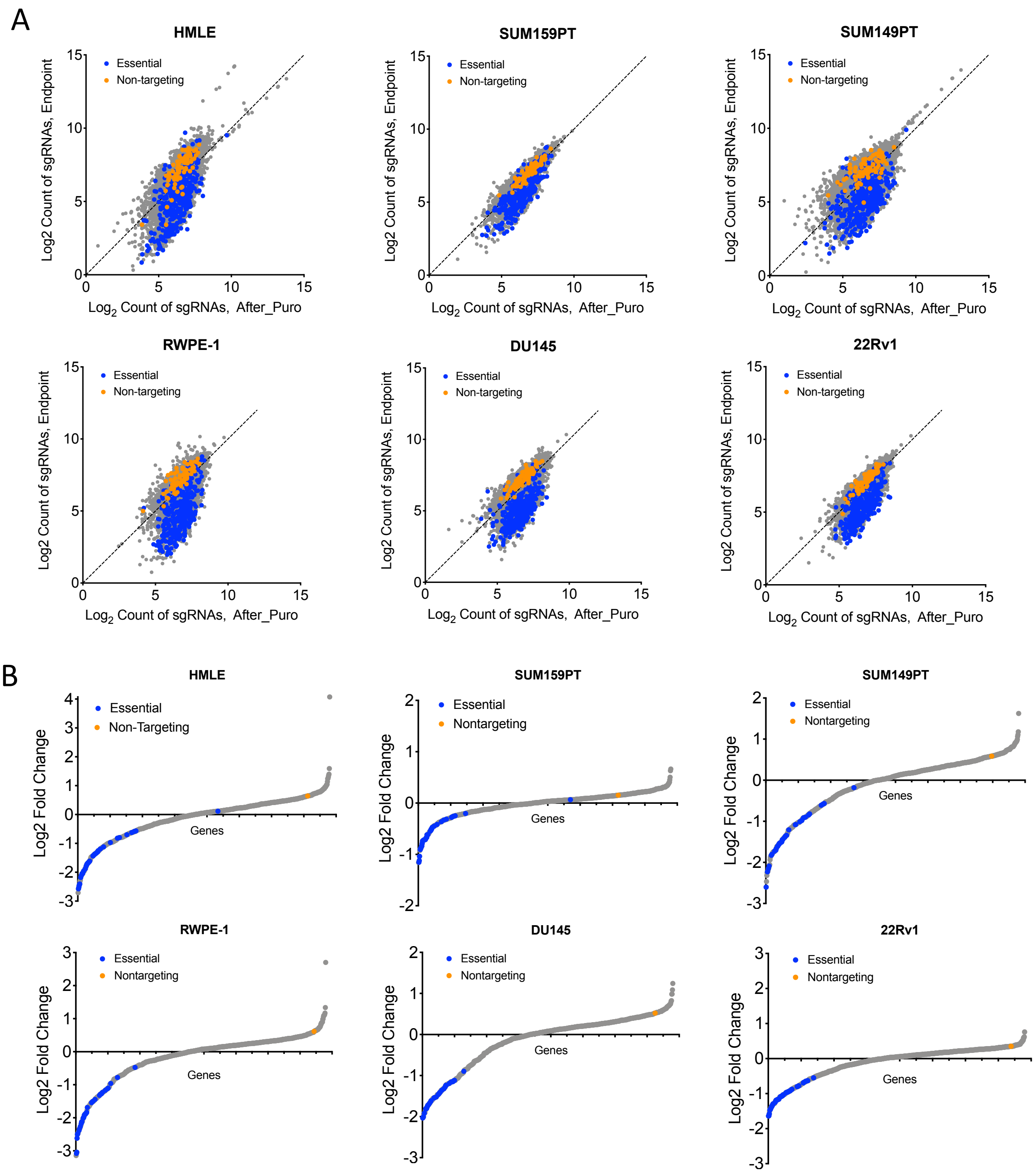

Supplementary Figure 2. sgRNA level log2 count and gene-level waterfall plots for EPIKOL screens on each cell line. *A.* Log2 counts of sgRNAs at initial and final time points in TNBC and prostate cells lines. ***B.*** Waterfall plots for Log$_2$ fold changes of genes after screening with EPIKOL for at least 15 population doublings in TNBC and prostate cell lines.

Depleted in p<0.05 cut off				gRNA	Function
HMLE	MDA MB 231	SUM159PT	SUM149PT	PLGNT1	Non-targeting Mix
+	+		+	CDC16 g1<br>CDC16 g2	Positive Control (MDA)
+	+	+	+	RPL11 g1<br>RPL11 g2	Positive Control (MDA and SUM159)
+	+	+	+	TOP2A g1<br>TOP2A g2	Proof of concept
+	+	+	+	PRMT5 g1<br>PRMT5 g2	
+	+	+	+	FBL g1<br>FBL g2	HMT
+	+	+	+	HDAC3 g1<br>HDAC3 g2	HDAC
+	+		+	WDR82 g1<br>WDR82 g2	COMPASS (HMT)
+	+			PRPF31 g1<br>PRPF31 g2	CHD8 (MLL), MLL2/3, MLL4/WBP7
+	+		+	CHAF1B g1<br>CHAF1B g2	CAF-1
+	+	+	+	SFPQ g1<br>SFPQ g2	SIN3A Complex
+	+	+	+	ELP3 g1<br>ELP3 g2	POL2 Elongator (HAT)
+	+		+	ELP5 g1<br>ELP5 g2	
+	+	+		NAT10 g1<br>NAT10 g2	HAT activity
+	+	+	+	USP7 g1<br>USP7 g2	BCOR complex (PcG and PcG like)
+	+	+	+	CDK1 g1<br>CDK1 g2	
+	+	+	+	PPP4C g1<br>PPP4C g2	
+	+	+	+	HCFC1 g1<br>HCFC1 g2	NSL, COMPASS, MLL-HCF, CHD8, MLL2/3, COMPASS-like MLL1,2, MLL4/WBP7
+				KANSL1 g1<br>KANSL1 g2	NSL, CHD8, MLL2/3, MLL4/WBP7
+	+		+	TRRAP g1<br>TRRAP g2	SWR, PCAF, TFTC-HAT, NuA4, SAGA, NuA4-related complex, STAGA
+	+	+	+	RBBP4 g1<br>RBBP4 g2	NuRF, SWI/SNF_Brg1(I), SWI/SNF_Brg1(II), SWI/SNF_Brm, NuRD,
	+	+	+	SS18L2 g1<br>SS18L2 g2	chromatin remodeling
	+	+	+	KDM2A g1<br>KDM2A g2	KDM
	+	+	+	ASH2L g1<br>ASH2L g2	CHD8 (MLL), MLL2/3, COMPASS (HMT)
	+			LAS1L g1<br>LAS1L g2	CHD8 (MLL) , MLL2/3, MLL4/WBP7
	+	+	+	RUVBL1 g1<br>RUVBL1 g2	CHD8, Ino80, MLL2/3 (MLL), NuA4 (HAT), SRCAP, SWR
	+	+	+	SUPT16H g1<br>SUPT16H g2	
	+			EXOSC9 g1<br>EXOSC9 g2	RNA exosome
	+		+	GTF3C4 g1<br>GTF3C4 g2	General TF - HAT activity
	+			CIT g1<br>CIT g2	
		+	+	DPY30 g1<br>DPY30 g2	COMPASS, Menin-associated_HMT, MLL-HCF, CHD8, MLL2/3, COMPASS-like MLL1,2,
		+	+	RBBP5 g1<br>RBBP5 g2	COMPASS, Menin-associated_HMT, MLL-HCF, CHD8, MLL2/3, COMPASS-like MLL1,2,
	+		+	KAT8 g1<br>KAT8 g2	NSL, CHD8, MLL2/3, COMPASS-like MLL1,2, MLL4/WBP7
	+	+	+	KANSL2 g1<br>KANSL2 g2	NSL
	+	+	+	KANSL3 g1<br>KANSL3 g2	
	+		+	YEATS4 g1<br>YEATS4 g2	NuA4, NuA4-related complex, SRCAP
		+	+	VPS72 g1<br>VPS72 g2	NuA4, NuA4-related complex
	+		+	RUVBL2 g1<br>RUVBL2 g2	Ino80, SWR, NuA4, NuA4-related complex, CHD8, MLL2/3, MLL4/WBP7, SRCAP
	+			WDR5 g1<br>WDR5 g2	ATAC, NSL, RING2-L3MBTL2, COMPASS, Menin-associated_HMT, MLL-HCF, CHD8,
	+	+	+	DR1 g1<br>DR1 g2	ATAC
		+	+	NIPBL g1<br>NIPBL g2	Histone acetylation
	+	+	+	HINFP g1<br>HINFP g2	

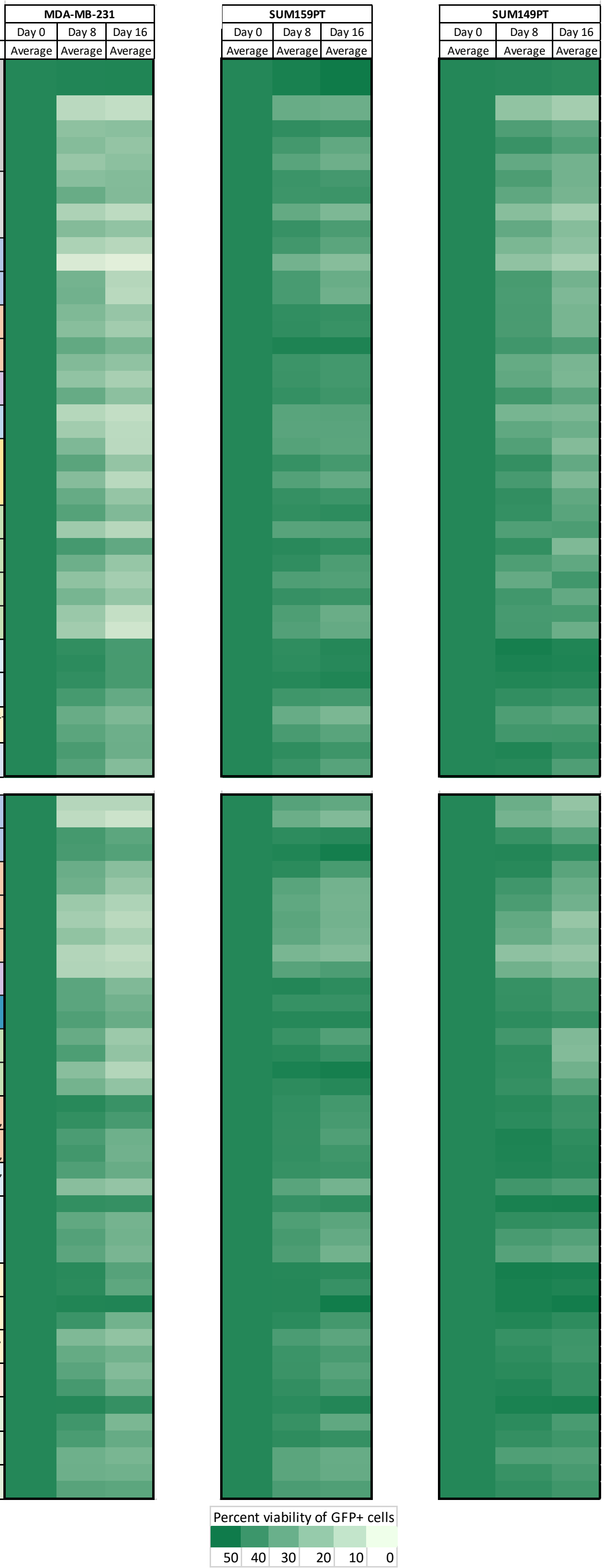

Supplementary Figure 3. Heat-map of competition assay results normalized to Day0 for TNBC cell lines. Left-hand side shows if a given gene is found in p<0.05 cutoff in each cell line. Complexes of the genes were indicated if applicable. Day8 and Day16 measurements of all sgRNAs were normalized to the corresponding Day0 measurement.

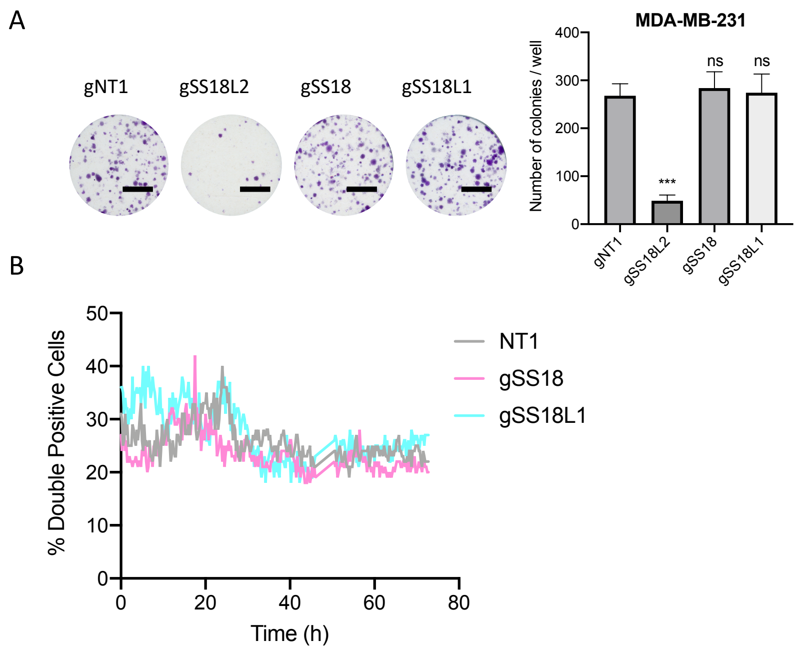

Supplementary Figure 4. Knockout of SS18 or SS18L1 does not decrease cell fitness or induce cell cycle arrest. *A.* Representative images of long-term colony formation assay and their statistical analysis for MDA-MB-231 cells infected with SS18L2, SS18 and SS18L1 sgRNAs. P values determined by two-tailed Student's *t*-test in comparison to NT1; *P<0.05, **P<0.01, ***P<0.001. Scale bar: 10 mm. ***B.*** Percentages of cells that express both mVenus and mCherry as an indicative of cells in G2/M phase upon SS18 and SS18L1 knockout.

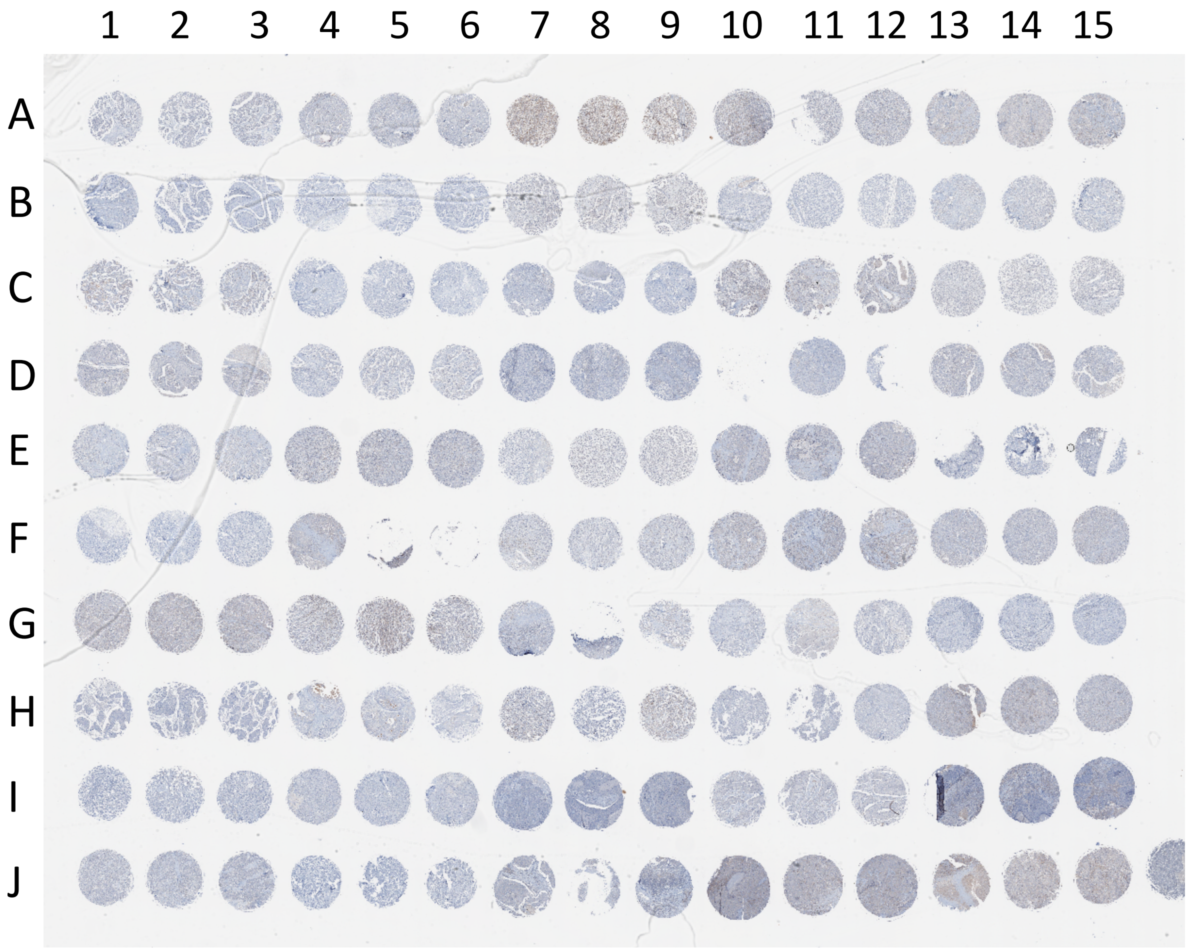

Supplementary Figure 5. Breast tissue microarray results stained with anti-SS18L2 antibody.

2- SUPPLEMENTARY MATERIALS & METHODS

Cloning of EPIKOL. All sgRNAs were synthesized as pooled oligonucleotides (LC Biosciences). Lyophilized oligonucleotides were resuspended and amplified by PCR with following conditions: For 50 µl of total PCR mix, 10 µl of 5X HF buffer (NEB,USA), 1 µl of dNTP mix (10 mM) (Thermo Fisher,USA) , 2.5 µl of forward primer (10 µM), 2.5 µl of reverse primer (10 µM), 0.1 µl of resuspended oligomix, 0.5 µl of Phusion High-fidelity DNA polymerase (NEB,USA), 33.4 µl of dH_2O were added. Thermal cycler conditions were: 30 sec at 98°C for initial denaturation, followed by 20 cycles of (10 sec at 98°C, 20 sec at 63°C, 15 sec at 72°C), 3 min at 72°C for final extension. Two tubes of 50 µl reaction were run on Agarose gel and correct sized bands were gel-extracted. 10 µg of lentiviral backbones lentiCRISPRv2 (Addgene #52961) and lentiGuide-Puro (Addgene #52963) were digested with BsmBI at 55°C for 6 h followed by agarose gel extraction. PCR-amplified oligos and gel-extracted vector backbones were purified with AMPure XP magnetic beads according to manufacturer's instructions. For ligation reaction, 100 ng of purified backbone, 15-20 ng of purified oligomix, 5 µl of Gibson assembly mastermix (NEB) and dH_2O up to 10 µl were mixed and incubated at 50°C for 1 h. 1 µl from ligation reaction was added onto 25 µl of electrocompetent cells (Lucigen) and cells were transformed by using Electroporator (Bio-Rad MicroPulser) according to manufacturer's instructions. 1 ml of recovery medium was added on cells immediately after pulse and incubated at 32°C for 1 h. 10 µl of bacteria culture were taken to 90 µl of LB and serial dilution was performed to estimate library coverage. Rest of the culture (~980 µl) was added directly on 500 ml of liquid LB containing ampicillin. All cultures were incubated overnight (12-15h) at 32°C. Next day, plasmid extractions were performed by using NucleoBond Xtra Midi kit (Macherey-Nagel). To ensure coverage, six electroporations for LentiCRISPRv2 and three electroporations for LentiGuide-Puro were performed and mixed after plasmid extraction which yielded 500x and 800x library coverage, respectively.

PCR amplifications from plasmid DNA. To determine sgRNA distribution in plasmid pools (LentiCRISPRv2 or LentiGuide-Puro), plasmid DNA (pDNA) were amplified by adding Illumina compatible sequences. 10 ng template DNA were mixed with 0.5 µl Phusion High-Fidelity DNA Polymerase (NEB, USA), 10 µl 5x GC Buffer (NEB, USA), 1 µl dNTP mix (10 mM each) (Thermo Fisher,USA), 2 µl Forward Stagger Mix (10 µM) and 2 µl Reverse Index Primer (10 µM) specific to each vector backbone and Nuclease-free water (NEB,USA) up to 50 µl. Thermal cycler conditions were as follows: denaturation for 30 s at 98°C, followed by (10 s at 98°C, 15 s at 63°C, 20 s at 72°C) for 16 cycles, final extension for 4 mins at 72°C. PCR amplicons were gel extracted using NucleoSpin Gel and PCR clean-up (Macherey-Nagel,

Germany) kit according to manufacturer's instructions and quantified using Nanodrop. Next generation sequencing was performed at Genewiz (USA) by using Hiseq (Illumina) with at least 10 million reads/plasmid library.

Cell culture. MDA-MB-231, SUM159PT and SUM149PT TNBC cell lines, immortalized human breast epithelial cells (HMLE) and HEK293T cells were kind gifts from Robert Weinberg (MIT, Boston, USA) [1]. LNCaP, 22Rv1, DU145 and RWPE-1 cells were purchased from ATCC. MDA-MB-231 and HEK293T cells were cultured in DMEM (Gibco,USA) supplemented with 10% fetal bovine serum (Gibco, USA) and 1% Penicillin/Streptomycin (Gibco,USA). SUM159PT and SUM149PT cell lines were cultured in Ham's F12 nutrient mix (Gibco, USA) supplemented with 5% FBS, 5 µg/ml insulin (Sigma-Aldrich, USA), 1 µg/ml hydrocortisone (Sigma-Aldrich, USA) and 10 mM HEPES (Thermo Fisher, USA). HMLE cells were cultured in MEGM medium as described [2]. All prostate cell lines except RWPE-1 were cultured in RPMI 1640 (Gibco, USA) supplemented with 10% fetal bovine serum (Gibco, USA) and 1% penicillin/streptomycin (Gibco, USA). RWPE-1 cells were cultured in Keratinocyte SFM media (Gibco, USA) supplemented with 0.05 mg/mL bovine pituitary extract and 5 ng/mL human recombinant epidermal growth factor (Gibco, USA). Cells were maintained in a humidified incubator at 37°C with 5% CO_2 level. All cell lines were tested regularly for mycoplasma infection.

Virus production, concentration and titration. For lentiviral packaging of EPIKOL, either Fugene 6 (Roche Applied Science) or Transporter 5 transfection reagents were used. For Fugene 6 transfection, HEK293T cells were plated as 2.5 x 10^6 cells per 10cm plate. Next day, 2500 ng EPIKOL (either in LentiCRISPRv2 or LentiGuide-Puro backbones), 2250 ng psPAX2 (Addgene 12260) and 225 ng VSV-G (Addgene 8454) plasmids were mixed in 200 µL serum-free DMEM. DNA mixture was then added into 200 µL serum-free DMEM containing 15 µL Fugene 6. For Transporter 5 transfection, 5x10^6 HEK293T cells were seeded onto 10cm plates. The media were refreshed with low FBS media (2% FBS in DMEM) at least 2 hours prior to transfection. For transfection, 6 µg plasmid DNA was mixed with 5.4 µg of CMV-8.2dVPR and 0.6 µg of CMV-VSV-g plasmids in 0.8 mL of 150 mM NaCl solution. 36 µL of Transporter 5 was added on top of the mixture and gently mixed. In both transfection types, mixtures were incubated for 30 minutes and distributed dropwise to 10 cm plates. Next day, transfection media were replaced by 8 ml fresh media per plate. Supernatant containing viral particles was collected 48 h and 72 h post-transfection and filtered through 45-µm filters [3]. To obtain concentrated viruses, supernatants were mixed with PEG8000 (Sigma-Aldrich, USA) (dissolved in PBS as 50% (w/v)) in 10% final concentration for overnight at 4°C. Next day, supernatants were centrifuged at 2500 rpm for 20 min at 4°C and pellets were resuspended in

PBS as 100x concentrated [3]. The viral aliquots were kept in -80°C until usage. Viral titers were determined on the cell line of interest. Briefly, cells were seeded as 2x10^5 cells per well of a 6-well plate and next day incubated with 10, 1, 10^{-1}, 10^{-2} µL viral supernatant in the presence of 8 µg/ml protamine sulphate (Sigma-Aldrich, USA) overnight. Next day, media with viral supernatants were replaced by fresh media. 36 hours later, each well of 6 well-plate were transferred to a 10cm plate with previously determined concentrations of puromycin (Sigma-Aldrich, USA), for that cell line for 3 days. Once the cells in uninfected wells were completely eliminated with puromycin, remaining cells in other wells were compared to uninfected/unselected parental controls. The viral volume that results in 30-50% transduction efficiency was used for the downstream experiments. For EPIKOL in LentiGuide-puro backbone, LentiCas9-blast (Addgene 52962) viruses were produced in the same manner.

Area Under the Curve Analysis. Library performance was evaluated using Area Under the Curve (AUC) calculations of the predefined sgRNA groups, such as 'Essential', 'Non-Essential' and 'Non-Targeting' [4]. The same method was used to evaluate the performance of EPIKOL. AUC calculation was run using the python code (https://github.com/mhegde) and by following the instructions on GitHub. EPIKOL library specific 'Input File' and 'ChiP File' were prepared, meanwhile sgRNAs were grouped as 'Targeting', 'Essential', 'Non-Targeting' and used for preparation of 'Gene Set' files. All results from the AUC analysis were plotted and analyzed in GraphPad Prism 8.

Genomic DNA isolation and Nested PCR. Genomic DNA (gDNA) was isolated by NucleoSpin Tissue kit (Macherey-Nagel, Germany) for TNBC cell lines and by PureLink Genomic DNA mini kit (ThermoFischer K-1820) for PCa cell lines according to manufacturer's instructions. For PCR amplification of TNBC gDNAs (external PCR), input gDNA amount was calculated as 250x coverage of the EPIKOL library which corresponded to 13.2 µg per sample (assuming 6.6 pg DNA per cell). For each sample, 13.2 µg gDNA was divided into four PCR tubes with 3.3 µg gDNA per 100 µl reaction. In external PCR, 3.3 µg gDNA, 1 µl Phusion High-Fidelity DNA Polymerase (NEB, USA), 20 µl 5x GC Buffer (NEB, USA), 2 µl dNTP mix (10 mM each) (Thermo Fisher,USA), 5 µl Forward External Primer (10 µM), 5 µl Reverse External Primer (10 µM) and Nuclease-free water (NEB,USA) up to 100 µl were mixed on ice. Thermal cycler conditions were as follows: denaturation for 3 mins at 95°C, followed by (25 s at 95°C, 20 s at 65°C, 15 s at 72°C) for 17 cycles, final extension for 3 mins at 72°C. PCR reactions were then combined. For internal PCR, 5 µl from combined PCR products were used as a template with 5 µl Forward Stagger Mix (10 µM) and 5 µl Reverse Index Primer (10 µM) in a 100ul reaction. Thermal cycler conditions were the same as external PCR except that

amplification was carried out for 23 cycles instead of 17. Final amplicons from duplicate internal PCRs were gel extracted using NucleoSpin Gel and PCR clean-up kit according to manufacturer's instructions and quantified using Nanodrop. All primer sequences are available in the **Supplementary Table 2**. Next generation sequencing was performed at Genewiz (USA) with at least 10 million reads/sample.

For library preparation of PCa samples, 4 µg of genomic DNA in total was amplified by using Kapa HiFi HotStart ReadyMix (Roche KK2602). For external PCR step, 8 reactions were prepared in 25 µL reaction volume using 0.5 µg of genomic DNA with 12.5 µL of Kapa HiFi HotStart ReadyMix, 2.5 µL of external forward primer (10 µM), 2.5 µL of external reverse primer (10 µM) and nuclease free water up to 25 µL. External PCR products for each sample were pooled to be used in internal PCR. For internal PCR, 2 reactions were prepared in 50 µL reaction volumes with 1 µL of pooled external PCR product, 25 µL of Kapa HiFi HotStart ReadyMix, 2.5 µL of mixed forward staggered primer pool (10 µM), 2.5 µL of indexed reverse primer (10 µM) and 19 µL of nuclease free water. PCR reactions were performed in the following conditions: initial denaturation at 95°C for 3 mins, denaturing at 95°C for 25 sec, annealing at 65°C for 20 sec, extension at 72°C for 15 sec. Final extension was performed at 72°C for 3 mins. The number of PCR cycles were 25x for external PCR and 15x for internal PCR.

Screen Analysis. MAGeCK algorithm (version 0.5.8) was used to identify significantly changed sgRNAs in knockout screens [5]. Reads from paired-end fastq files were counted at the sgRNA level and normalized to library size. Biological replicates were presented as individual input files during sgRNA counting. Individual counts were combined as one output count for each sgRNA in every condition, with median normalization to obtain gene level log fold changes. A $p<0.05$ cutoff was applied to the gene-level analysis to identify significantly depleted genes. sgRNA counts were also normalized as Read Per Million (RPM) and converted to Log_2 values [4, 6]. Kernel density estimation (KDE) plots of the Log_2 transformed sgRNA counts were plotted with R using the geom_density function in the *ggplot2* package. Pearson correlations were calculated and plotted with R using the pairs.panels function in the *psych* package. Cumulative density plots were plotted with the stat_ecdf function in *ggplot2*. Epigenetic complex-based gene sets were curated into .gmt files with the information obtained from the Epifactors database and a comprehensive literature review. Base file of the epigenetic complex-based gene sets can be found in **Supplementary Table 3**.

Clonogenic Assays. To assess relative cell fitness, control cells (LentiGuide-NT1 infected) and cells carrying sgRNAs against candidate genes were seeded as 750 cells/well in triplicates in 6-well plates. Cells were allowed to grow for 12 days; media were changed regularly. At the end of the incubation period, media were discarded, cells were washed with PBS and fixed with ice-cold 100% methanol for 5 minutes. Methanol was discarded and cells were stained with crystal violet for 15 minutes. Counting of colony numbers was performed by using ImageJ with the same threshold value for each well.

Western Blotting. Western blots were performed as described [7]. Briefly, cells together with the media that they were growing in were harvested and centrifuged. Primary antibodies used were GAPDH (Abcam ab9485, USA), PARP (Abcam, ab74290, USA). Secondary antibody was goat anti-rabbit (Abcam ab97051, USA) conjugated to HRP.

Annexin V Staining. Annexin V staining was performed with Muse® Annexin V & Dead Cell Kit (Luminex, MCH100105) according to the manufacturer's instructions. Briefly, MDA-MB-231 cells were collected 9 days post-transduction and adjusted to be 300-500 cells/µl per gene for the measurements. Cells were centrifuged at 1200 rpm for 5 minutes. The supernatant was removed, and pellet was resuspended in 500 µl of cold PBS with 1% FBS. Cell suspension was centrifuged again and resuspended in 75 µl of cold PBS with 1% FBS and mixed with 75 µl of Annexin V & Dead Cell Reagent. Samples were incubated at room temperature for 20 minutes and analyzed with Muse Cell Analyzer (Merck, Darmstadt, Germany) with 5000 events per sample. Gates were determined according to parental cells.

Cell Cycle Analysis. $1x10^6$ MDA-MB-231 cells were collected 9 days post-transduction and centrifuged at 1200 rpm for 5 minutes. The supernatant was removed, and the pellet was resuspended in 1 ml PBS. Samples were centrifuged at 1200 rpm for 5 minutes. Most of the supernatant was removed and the pellet was resuspended in PBS. Cell suspension was added drop by drop into the freshly prepared, cold 1 ml 70% Ethanol while vortexing for fixation. Samples were kept in -20˚C for 24 hours. After incubation, 200 µl of fixed cells were transferred into 15 ml conical centrifuge tube and following two rounds of wash by centrifugation at 1200 rpm for 5 minutes at room temperature, cells were resuspended in 150 µl of Muse Cell Cycle Reagent (The Muse® Cell Cycle Kit (Luminex, MCH100106)). Samples were incubated with the reagent at room temperature for 30 minutes in the dark and run through the Muse Cell Analyzer (Merck, Darmstadt, Germany) with 10,000 events per sample and analyzed with the Muse Cell Analyzer software. Gates were determined according to parental cells.

Immunohistochemistry and scoring. Breast cancer tissue microarray was purchased from US Biomax (BR1509). Immunohistochemistry (IHC) was performed to assess SS18L2 expression levels using SS18L2 primary antibody (rabbit polyclonal, HPA047377, Sigma-Aldrich). The Discovery Ultra autostainer (Ventana) was used. Slides were deparaffinized. Antigen retrieval was conducted with Tris-based solution (CC1, Ventana) for 64 min at 95°C. Primary antibody was applied for 16 min at room temperature as 1:500 in Discovery Ab Diluent (Ventana). Detection was conducted with UltraMap DAB anti-Rb Detection Kit (Ventana). Slides were counterstained and mounted. Slides were then imaged with the Aperio AT2 Scanner (Leica Biosystems). Images were digitally scored by a pathologist (Dr. L Fazli) with the Positive Pixel Count Algorithm on Aperio ImageScope (Leica Biosystems). Expression levels of tumor cores were determined as follows depending on the calculated digital score: weak/negative if below 1, moderate if between 1 and 5.5, strong if more than 5.5. All information about cores and SS18L2 scores can be found in **Supplementary Table 4**.

RNA sequencing and transcriptome analysis. Total RNAs were isolated by using MN Nucleospin RNA isolation kit according to manufacturer's instructions. Library preparation and sequencing was performed at University of Oxford (Oxford, UK). Briefly, RNA was DNase I-treated, cleaned and concentrated (Zymo RNA Clean and Concentrator, Zymo Research) then enriched for poly(A) mRNA (NEBNext poly(A) mRNA Magnetic Isolation Module, NEB Biosystems, Ipswich, UK). Sequencing libraries were prepared using the NEBNext Ultra II RNA Library Prep kit (New England Biolabs). RNA quality was assessed using High Sensitivity RNA Screentape and an Agilent 4200 tapestation. Single-indexed and multiplexed samples were run on an Illumina Next Seq 500 sequencer using a NextSeq 500 v2 kit (FC-404–2005; Illumina, Can Diago, CA) for paired-end sequencing. Sequencing length was 42 bp. Bioinformatic analysis of the samples were performed to identify differentially expressed genes after pre-processing of the sequencing data. fastQC was used to assess the quality of the sequencing reads for each sample. The sequencing data was then aligned to the GENCODE human transcriptome reference (GRCh38) using STAR [8]. The output from STAR was sorted by co-ordinate transcriptome BAM file. The average alignment rate amongst all samples was 85.9%. Further quality control of the sample BAM files was performed using RSeQC [9] and Picard, with several quality metrics collected for assessment and comparison using MultiQC [10]. SALMON was then used to quantify the transcripts for each sample based on the Transcripts Per Million (TPM) method [11]. The SALMON transcript quantification files are read and summarized at the gene-level using the R package *tximport* [12]. *DESeq2* was used to identify differentially expressed genes between cells infected with NT1 carrying or SS18L2 sgRNA carrying lentiviruses [13]. *DESeq2* performs a median of ratios method for normalization of the counts and applies a negative-binomial generalized linear model with a

Wald test to analyze each gene between the two groups of treated cells. Differentially expressed genes (DEGs) were defined with a threshold for Log_2FoldChange>1 (up-regulated) or Log2FoldChange<-1 (down-regulated) and false discovery rate (FDR)<0.01. Gene set enrichment analysis was performed with Log2FoldChange rank-ordered gene lists by using GSEA software and all available gene sets from MsigDB [14].

Quantitative RT-PCR. RNA isolation and cDNA synthesis were performed as described [15].List of primers can be found in **Supplementary Table 2**.

3- REFERENCES

1. Elenbaas, B., et al., *Human breast cancer cells generated by oncogenic transformation of primary mammary epithelial cells.* Genes Dev, 2001. **15**(1): p. 50-65.
2. Bierie, B., et al., *Integrin-beta4 identifies cancer stem cell-enriched populations of partially mesenchymal carcinoma cells.* Proc Natl Acad Sci U S A, 2017. **114**(12): p. E2337-E2346.
3. Onder, T.T., et al., *Chromatin-modifying enzymes as modulators of reprogramming.* Nature, 2012. **483**(7391): p. 598-602.
4. Sanson, K.R., et al., *Optimized libraries for CRISPR-Cas9 genetic screens with multiple modalities.* Nat Commun, 2018. **9**(1): p. 5416.
5. Li, W., et al., *MAGeCK enables robust identification of essential genes from genome-scale CRISPR/Cas9 knockout screens.* Genome Biol, 2014. **15**(12): p. 554.
6. Breslow, D.K., et al., *A CRISPR-based screen for Hedgehog signaling provides insights into ciliary function and ciliopathies.* Nat Genet, 2018. **50**(3): p. 460-471.
7. Senbabaoglu, F., et al., *Identification of Mitoxantrone as a TRAIL-sensitizing agent for Glioblastoma Multiforme.* Cancer Biol Ther, 2016. **17**(5): p. 546-57.
8. Dobin, A., et al., *STAR: ultrafast universal RNA-seq aligner.* Bioinformatics, 2013. **29**(1): p. 15-21.
9. Wang, L., S. Wang, and W. Li, *RSeQC: quality control of RNA-seq experiments.* Bioinformatics, 2012. **28**(16): p. 2184-5.
10. Ewels, P., et al., *MultiQC: summarize analysis results for multiple tools and samples in a single report.* Bioinformatics, 2016. **32**(19): p. 3047-8.
11. Patro, R., et al., *Salmon provides fast and bias-aware quantification of transcript expression.* Nat Methods, 2017. **14**(4): p. 417-419.
12. Soneson, C., M.I. Love, and M.D. Robinson, *Differential analyses for RNA-seq: transcript-level estimates improve gene-level inferences.* F1000Res, 2015. **4**: p. 1521.
13. Love, M.I., W. Huber, and S. Anders, *Moderated estimation of fold change and dispersion for RNA-seq data with DESeq2.* Genome Biol, 2014. **15**(12): p. 550.
14. Subramanian, A., et al., *GSEA-P: a desktop application for Gene Set Enrichment Analysis.* Bioinformatics, 2007. **23**(23): p. 3251-3.
15. Ebrahimi, A., et al., *Bromodomain inhibition of the coactivators CBP/EP300 facilitate cellular reprogramming.* Nat Chem Biol, 2019. **15**(5): p. 519-528.
