# Supplementary material for: EPIKOL, a chromatin-focused CRISPR/Cas9-based screening platform, to identify cancer-specific epigenetic vulnerabilities
Source: Cell Death Dis. 2022 Aug 16;13(8):710. doi: 10.1038/s41419-022-05146-4 (PMC9381743; doi:10.1038/s41419-022-05146-4)
Supplement: Supplementary file 3 — FULL LENGTH WESTERN BLOT IMAGES [file 41419_2022_5146_MOESM3_ESM.pdf]

FULL BLOT IMAGES

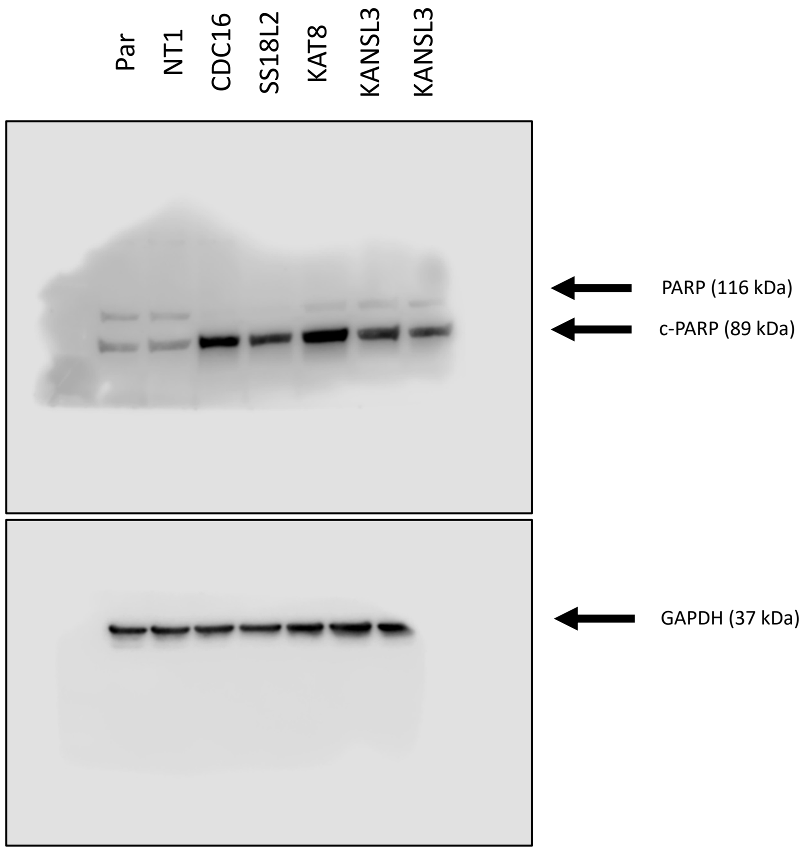

BLOT RESULT SHOWN IN MANUSCRIPT

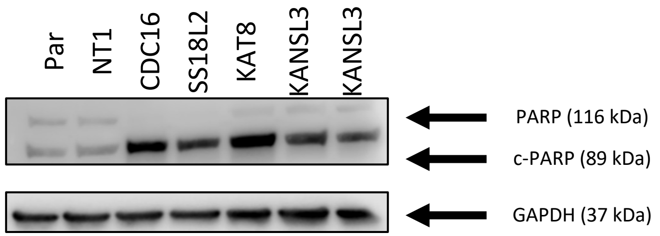

SuperSignal™ West Femto Maximum Sensitivity Substrate (Thermo Scientific, 34095, USA) was used for signal detection for PARP blot

Pierce ™ ECL Western Blotting Substrate (Thermo Scientific, 32106, USA) was used for signal detection for GAPDH blot
